# VgrG and PAAR Proteins Define Distinct Versions of a Functional Type VI Secretion System

**DOI:** 10.1371/journal.ppat.1005735

**Published:** 2016-06-28

**Authors:** Francesca R. Cianfanelli, Juliana Alcoforado Diniz, Manman Guo, Virginia De Cesare, Matthias Trost, Sarah J. Coulthurst

**Affiliations:** 1 Division of Molecular Microbiology, School of Life Sciences, University of Dundee, Dundee, United Kingdom; 2 Medical Research Council Protein Phosphorylation and Ubiquitylation Unit, School of Life Sciences, University of Dundee, Dundee, United Kingdom; Centre National de la Recherche Scientifique, Aix-Marseille Université, FRANCE

## Abstract

The Type VI secretion system (T6SS) is widespread among bacterial pathogens and acts as an effective weapon against competitor bacteria and eukaryotic hosts by delivering toxic effector proteins directly into target cells. The T6SS utilises a bacteriophage-like contractile machinery to expel a puncturing device based on a tube of Hcp topped with a VgrG spike, which can be extended by a final tip from a PAAR domain-containing protein. Effector proteins are believed to be delivered by specifically associating with particular Hcp, VgrG or PAAR proteins, either covalently (‘specialised’) or non-covalently (‘cargo’ effectors). Here we used the T6SS of the opportunistic pathogen *Serratia marcescens*, together with integratecd genetic, proteomic and biochemical approaches, to elucidate the role of specific VgrG and PAAR homologues in T6SS function and effector specificity, revealing new aspects and unexpected subtleties in effector delivery by the T6SS. We identified effectors, both cargo and specialised, absolutely dependent on a particular VgrG for delivery to target cells, and discovered that other cargo effectors can show a preference for a particular VgrG. The presence of at least one PAAR protein was found to be essential for T6SS function, consistent with designation as a ‘core’ T6SS component. We showed that specific VgrG-PAAR combinations are required to assemble a functional T6SS and that the three distinct VgrG-PAAR assemblies in *S*. *marcescens* exhibit distinct effector specificity and efficiency. Unexpectedly, we discovered that two different PAAR-containing Rhs proteins can functionally pair with the same VgrG protein. Showing that accessory EagR proteins are involved in these interactions, native VgrG-Rhs-EagR complexes were isolated and specific interactions between EagR and cognate Rhs proteins identified. This study defines an essential yet flexible role for PAAR proteins in the T6SS and highlights the existence of distinct versions of the machinery with differential effector specificity and efficiency of target cell delivery.

## Author Summary

Pathogenic bacteria use a biological nanomachine called the ‘Type VI secretion system’ (T6SS) to fire toxic proteins into other cells. These target cells can be rival bacterial cells, allowing the T6SS-wielding bacteria to outcompete other pathogens or harmless bacteria, or cells of a human, animal or plant, to help the bacteria cause an infection. The T6SS can deliver multiple different toxins, known as ‘effectors’, into target cells. This work aimed to learn more about how the machinery is able to recruit effectors and fire them from the bacterial cell into the target cell. To do this, we used a T6SS in *Serratia marcescens*, a pathogen causing hospital-acquired infections, which kills competitor bacteria very efficiently and is a good model for other T6SSs. We found that recently-identified components of the T6SS, called PAAR proteins, are an essential part of the machinery and also that several different versions of the machinery can form. These versions are able to fire different subsets of effectors and to deliver effectors into target cells with different efficiency. Our results help us to better understand how the T6SS machinery is able to deploy so many different toxins and kill competitor bacterial cells so effectively.

## Introduction

Bacteria utilise a variety of mechanisms to deliver specific proteins to the extracellular environment or directly into a target cell, processes collectively termed protein secretion. Bacterial protein secretion systems play a critical role in pathogenicity and can also be instrumental for interaction with other bacteria and the environment. To date, six major secretion systems have been described in Gram-negative bacteria (Types I-VI) [1]. The Type VI secretion system (T6SS) is widely distributed and has been linked with pathogenicity and/or interaction with eukaryotic cells in a number of important bacterial pathogens. These include *Pseudomonas aeruginosa*, *Vibrio cholerae* and *Burkholderia* species, where anti-eukaryotic effector proteins delivered into target host cells by the T6SS have been described [2–8]. However, it is becoming clear that many, probably most, T6SSs are used as highly efficient weapons in competition against rival bacteria. Such ‘anti-bacterial’ T6SSs have been described in varied species, including *P*. *aeruginosa*, *V*. *cholerae*, *Serratia marcescens* and *Acinetobacter baumannii* [9–12]. They should play an important role in promoting pathogen survival and fitness in polymicrobial niches, including infection sites and environmental reservoirs [13]. Anti-bacterial T6SSs can simultaneously deliver a variety of anti-bacterial toxins (effectors) into target bacterial cells, whilst the secreting cell and its siblings are prevented from self-killing by possession of specific immunity proteins, each able to neutralise its cognate toxic effector [7, 13]. Anti-bacterial effectors identified to date include cell wall-degrading peptidoglycan hydrolases [14–17], cytoplasmic acting DNases [18–20] and membrane targeting toxins [21, 22].

T6SSs are complex molecular nanomachines formed by a minimum of 13 conserved ‘core’ components (TssA-M). According to current models [23–27], the core components assemble into a structure resembling an inverted bacteriophage tail anchored to the bacterial cell envelope through a membrane-associated complex. Contraction of a sheath composed of TssB and TssC results in ejection of a puncturing structure, comprising a tube of stacked hexamers of Hcp (TssD) topped by a spike-like trimer of VgrG (TssI), towards the target. PAAR repeat-containing proteins have recently been proposed to be an additional component of the T6SS machinery, binding the distal end of the VgrG trimer to complete the final sharp tip of the machinery and also acting as a site for effector recruitment [28]. However the precise role of PAAR proteins, including whether they are essential for T6SS functionality, is still unclear.

Recent work has shed light on how the T6SS is able to recruit and deliver diverse effector proteins, namely by decorating the expelled Hcp-VgrG-PAAR puncturing device with varied effectors that are simultaneously delivered into a target cell when the system ‘fires’. The current model [25, 29] suggests that an effector can either be fused to a homologue of one of the components of the Hcp-VgrG-PAAR structure as an additional domain (‘specialised’ effector), or can non-covalently interact with one of the components of this structure (‘cargo’ effector). Each effector is therefore thought to depend exclusively on a particular component for its specific delivery [30, 31]. Many examples of specialised effector domains fused to VgrG or PAAR proteins have been identified, including the C-terminal domains of PAAR-containing Rhs proteins [7]. Several small cargo effectors have been shown to interact with the interior of the cognate Hcp hexamer and several larger ones have been shown to interact with particular VgrG proteins [32–34], whilst others might interact with specific PAAR proteins [28]. Additionally, certain cargo and specialised effectors require an accessory protein to mediate interaction with the T6SS [18, 34–36].

*Serratia marcescens* is an opportunistic pathogen which can infect diverse organisms and is responsible for many hospital acquired infections [37, 38]. The strain *S*. *marcescens* Db10 possesses a single T6SS which displays potent anti-bacterial activity against both closely and distantly related competitors, fires ‘offensively’ without needing a cell-contact trigger, and provides a good model for studying the T6SS [12, 39, 40]. Multiple anti-bacterial effectors are delivered by this system, including the cargo peptidoglycan amidase effectors Ssp1 and Ssp2 (Tae4.1^SM^ and Tae4.2^SM^), the specialised effectors Rhs1 and Rhs2, and other cargo effectors of unknown function, Ssp3-Ssp6 [18, 41, 42]. In this study, we aimed to elucidate the roles of each VgrG homologue in T6SS function and specific effector delivery, and additionally to utilise our system to address the role and essentiality of PAAR proteins within the T6SS. Our results reveal important functional aspects and unexpected subtleties in effector recognition and delivery by the T6SS, allowing the current model to be refined. We present evidence that PAAR proteins are indeed essential components of the T6SS and show that specific VgrG-PAAR combinations are required to form functional T6SSs in *S*. *marcescens*. These combinations define three alternative VgrG-PAAR-based assemblies with differential specificity and efficiency in delivery of cargo and specialised effectors, even those effectors which do not display an absolute requirement for a particular VgrG. We also discovered that distinct PAAR-containing Rhs proteins can utilise the same VgrG, perhaps through their interaction with cognate EagR accessory proteins, further illuminating the ability of this secretion machinery to flexibly recruit and deploy a range of effector proteins.

## Results

### Role of the two VgrG homologues in the T6SS of *S*. *marcescens* Db10

**Fig 1:**
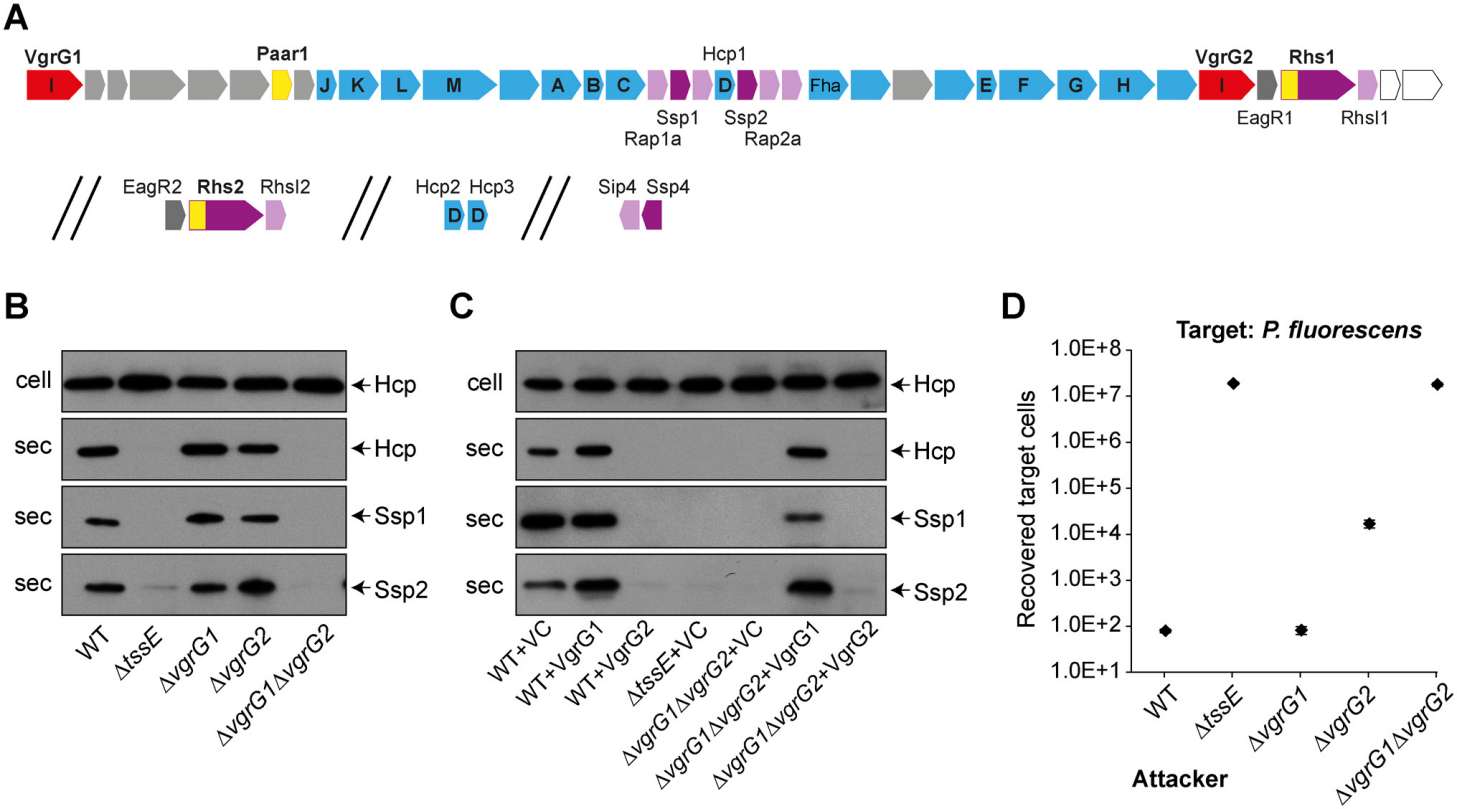
Differential requirement for the two VgrG proteins of *Serratia marcescens* for Type VI secretion system function and anti-bacterial activity. (A) Schematic depiction of the T6SS genetic loci in *S*. *marcescens* Db10 indicating the two VgrG homologues (red), PAAR proteins or PAAR repeat containing domains (yellow), effectors (violet), immunity proteins (pink) and other conserved T6SS components (blue; core TssA-TssM components are indicated by letter). (B) Immunoblot detection of Hcp1, Ssp1 and Ssp2 in cellular and secreted fractions of wild type (WT) or mutant (Δ*tssE*, Δ*vgrG1*, Δ*vgrG2* and Δ*vgrG1*Δ*vgrG2*) strains of *S*. *marcescens* Db10. The Δ*tssE* mutant has an inactive T6SS. (C) Immunoblot detection of secreted proteins in wild type or mutant strains carrying either the vector control plasmid (+VC, pSUPROM) or plasmids directing expression of VgrG1 (+VgrG1, pSC622) or VgrG2 (+VgrG2, pSC623) *in trans*. (D) Number of recovered cells of *P*. *fluorescens* 55 following co-culture with wild type or mutant strains of *S*. *marcescens* Db10 as attacker. Points show mean +/- SEM (n = 4).

The T6SS gene cluster of *S*. *marcescens* Db10 encodes two different VgrG homologues named VgrG1 (SMDB11_2244) and VgrG2 (SMDB11_2276) (Fig 1A, S1 Fig). Due to the lack of extended C-terminal domains, neither is predicted to be a specialised VgrG protein. T6SS-dependent secretion of both VgrGs has been observed previously [42], suggesting a role for both homologues in functionality of the machinery. To investigate the function of the two VgrG proteins, mutants carrying in-frame deletions of *vgrG1*, *vgrG2* or both genes were constructed, and the ability of these mutants to secrete Hcp1 and the cargo effector proteins Ssp1 and Ssp2 to the medium was monitored by immunoblotting. Loss of either individual VgrG homologue did not have a significant impact on T6SS-dependent secretion (Fig 1B). However, deletion of both VgrG genes resulted in a non-functional T6SS, confirming that it is essential to have at least one VgrG protein for the T6SS to fire. Expression of VgrG1 *in trans* restored the ability of the Δ*vgrG*1Δ*vgrG2* mutant to secrete Hcp1 and effectors. In contrast, plasmid-based expression of VgrG2 resulted in a complete abrogation of secretion even in the wild type background (Fig 1C). Next, the T6SS-dependent anti-bacterial ability of the VgrG mutants was assessed against *P*. *fluorescens* (Fig 1D). Co-culture of wild type and mutant strains of *S*. *marcescens* Db10 as ‘attackers’ with this ‘target’ organism revealed that, as expected, the Δ*vgrG*1Δ*vgrG2* mutant had lost all anti-bacterial activity. Consistent with the *in vitro* secretion assays, deletion of *vgrG1* did not result in any loss of anti-bacterial activity. However, in contrast, deletion of *vgrG2* alone caused a significant impairment in T6SS-dependent anti-bacterial activity (Fig 1D). An even greater reduction in antibacterial activity was observed against other targets, *S*. *marcescens* ATCC274 and *E*. *coli* (S1 Fig, part B). This result reveals that whilst the two VgrG homologues appear essentially redundant for basic T6SS activity (ability to secrete Hcp and cargo effectors to the medium), they are clearly not interchangeable for effective T6SS killing of target cells.

### VgrG2 is required for delivery of the specialised effectors Rhs1 and Rhs2 and plays a distinct role in cargo effector delivery

**Fig 2:**
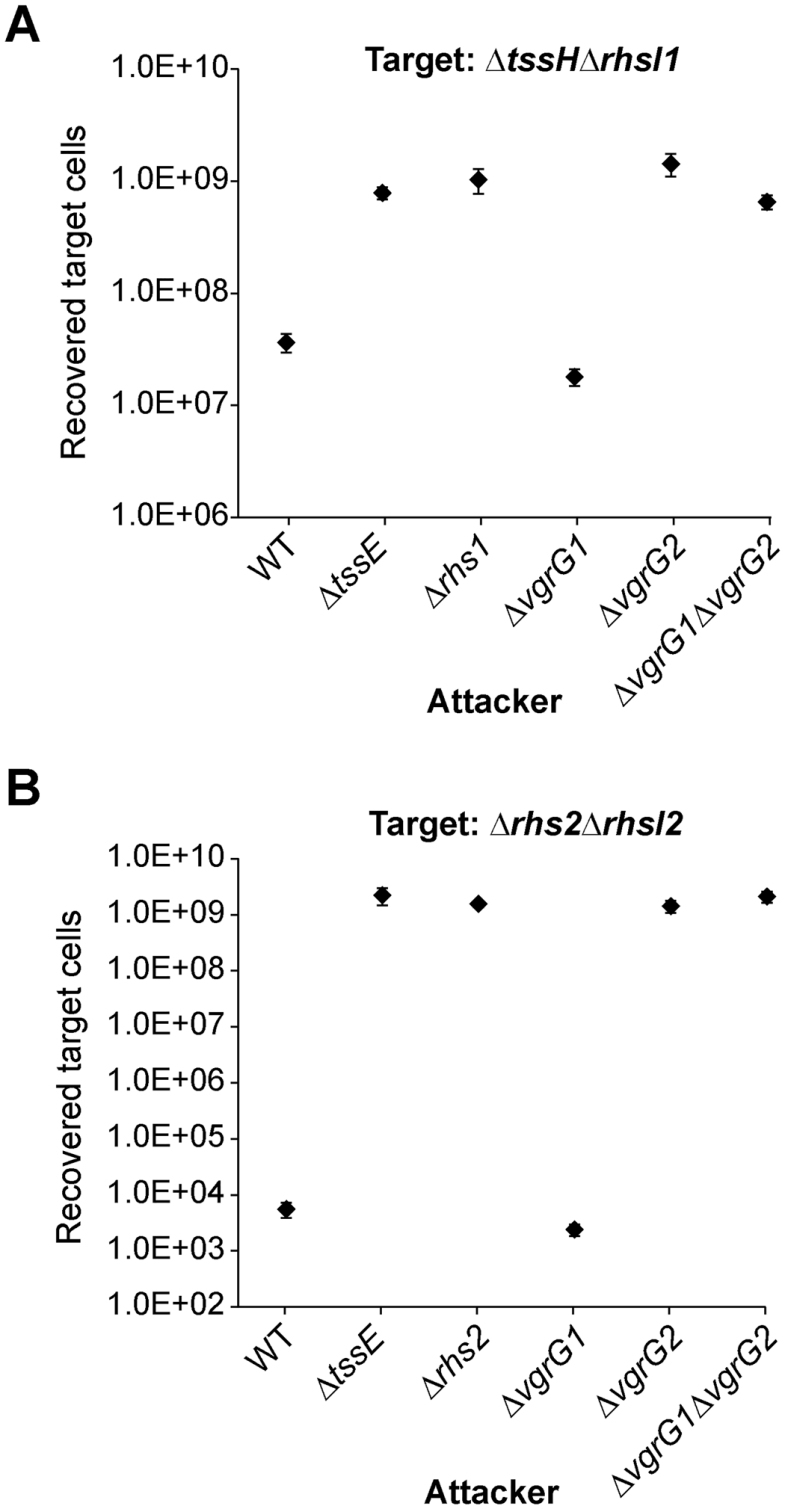
Rhs1 and Rhs2 depend exclusively on VgrG2 for their delivery into target cells. (A-B) Recovery of a target strain lacking *rhsI1* (*S*. *marcescens* Db10 Δ*tssH*Δ*rhsI1*), part A, or lacking *rhsI2* (*S*. *marcescens* Db10 Δ*rhs2*Δ*rhsI2*), part B, following co-culture with wild type (WT) or mutant (Δ*tssE*, Δ*rhs1*, Δ*rhs2*, Δ*vgrG1*, Δ*vgrG2* and Δ*vgrG1*Δ*vgrG2*) strains of Db10 as attacker, as indicated. Points show mean ± SEM (n = 4).

These results suggested that the two VgrG homologues are likely to be specifically responsible for delivering certain effectors. Indeed it has been reported recently that a particular VgrG protein can act as an indispensable carrier for a certain specialised (PAAR domain containing) or larger cargo effectors [30, 31, 34]. Previously, we have described two Rhs family proteins as specialised, PAAR-containing effectors delivered through the T6SS of *S*. *marcescens* Db10 [18]. We therefore decided to investigate the role of the two VgrGs in Rhs1 and Rhs2 delivery. If one particular VgrG is required for deployment of a specific effector, a mutant lacking that VgrG should be unable to exert any anti-bacterial activity against a target strain susceptible to the effector. Therefore the ability of the single and double VgrG mutants to act against target strains Δ*rhsI1* and Δ*rhsI2*, non-immune mutants of *S*. *marcescens* Db10 susceptible to Rhs1 and Rhs2 respectively, was tested. This revealed that both Rhs1 and Rhs2 required VgrG2 for their delivery, since a Δ*vgrG2* mutant attacker was unable to inhibit either target strain, behaving indistinguishably from a mutant lacking either a functional T6SS (Δ*tssE*) or the cognate Rhs effector, whilst neither effector required VgrG1 (Fig 2).

**Fig 3:**
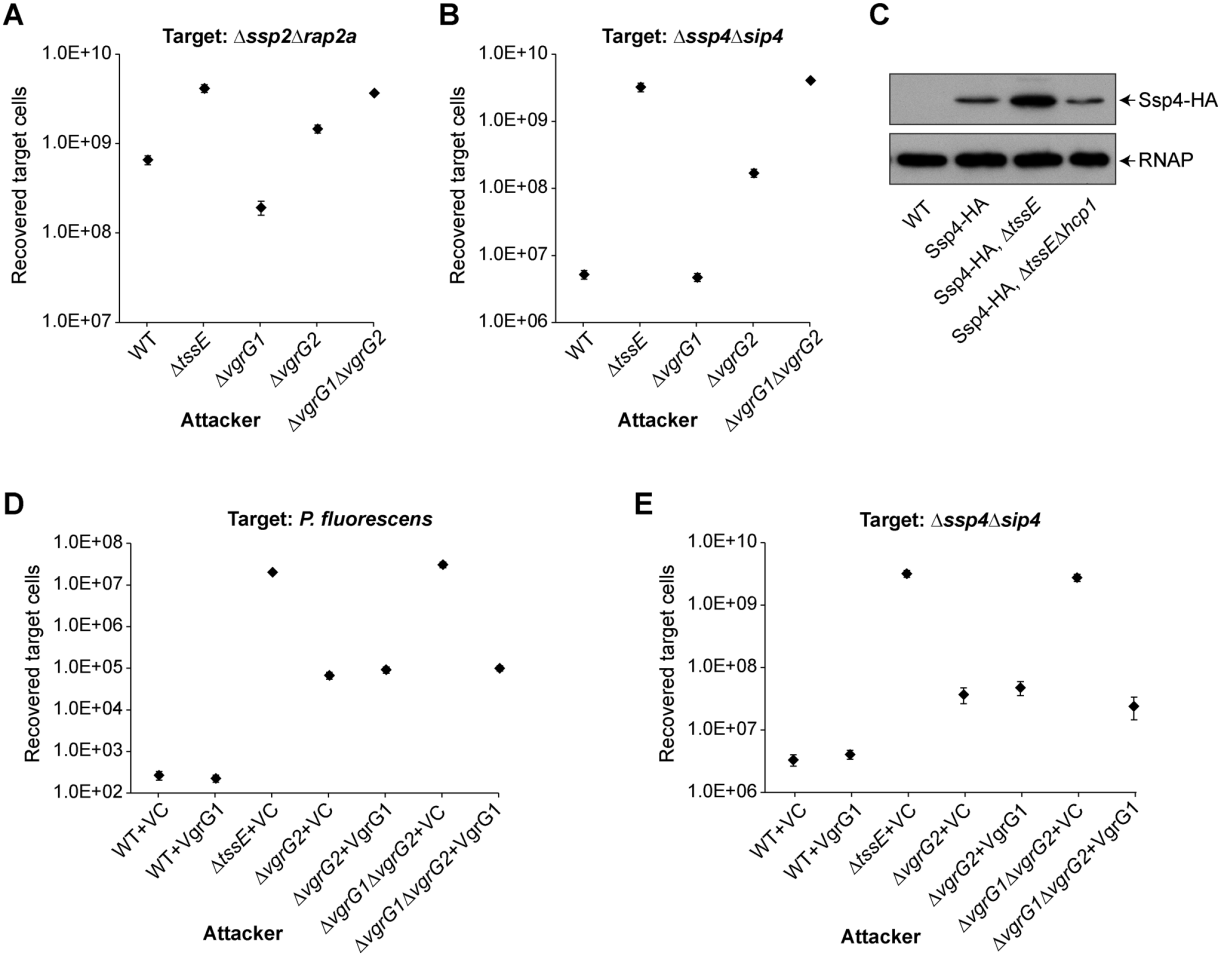
Hcp-dependent cargo effectors Ssp2 and Ssp4 display a preference for delivery by VgrG2, even in the presence of excess VgrG1. (A-B) Recovery of a target strain lacking *rap2a* (*S*. *marcescens* Db10 Δ*ssp2*Δ*rap2a*), part A, or lacking *sip4* (*S*. *marcescens* Db10 Δ*ssp4*Δ*sip4*), part B, following co-culture with wild type (WT) or mutant (Δ*tssE*, Δ*vgrG1*, Δ*vgrG2* and Δ*vgrG1*Δ*vgrG2*) strains of Db10 as attacker. Points show mean ± SEM (n = 4). (C) Anti-HA and anti-RNAP immunoblot of total cellular protein from wild type *S*. *marcescens* Db10 or strains expressing an Ssp4-HA fusion protein from the normal chromosomal location, either in a wild type background (Ssp4-HA) or in strains lacking TssE (Ssp4-HA, Δ*tssE*) or TssE and Hcp1 (Ssp4-HA, Δ*tssE*Δ*hcp1*). (D-E) Recovery of target organisms *P*. *fluorescens* 55, part D, or *S*. *marcescens* Db10 Δ*ssp4*Δ*sip4*, part E, following co-culture with wild type or mutant strains carrying either the vector control plasmid (+VC, pSUPROM) or a plasmid directing the expression of VgrG1 (+VgrG1, pSC622) *in trans*.

We also considered the contribution of individual VgrG proteins to delivery of small cargo effectors, using Ssp2 and Ssp4 as examples. Such effectors are proposed to be bound within the lumen of the Hcp hexamer, resulting in their stabilisation in the cytoplasm of the secreting cell and their ultimate secretion, and also to show no specific dependence on one VgrG over another [31, 33]. Unexpectedly, deletion of *vgrG2* resulted in a significant reduction, but not total loss, of T6SS-mediated anti-bacterial activity against target strains specifically susceptible to Ssp2 and Ssp4 (Fig 3A and 3B; differences between Δ*vgrG2* and the wild type or the T6SS mutant statistically significant, p < 0.01). Ssp2 is related to the amidase Tse1 which has been shown to depend on Hcp1 for intracellular stability and secretion in *P*. *aeruginosa* [31, 33]. To provide evidence that Ssp4 is an Hcp-dependent effector, we determined whether it is similarly stabilised or ‘chaperoned’ by one of the Hcp homologues in *S*. *marcescens*. Hcp1 is the major Hcp homologue in *S*. *marcescens* Db10 and is essential for secretion system activity and delivery of Ssp4, whereas Hcp2 and Hcp3 are not required or sufficient for T6SS activity or for Ssp4 delivery (S2 Fig). Therefore cellular levels of Ssp4 were examined in the wild type, a T6SS inactive strain (Δ*tssE*, where all the Ssp4 is retained in the cell) and a Δ*tssE*Δ*hcp1* mutant strain (where any loss of stability in the absence of Hcp1 will be observed). Using strains encoding an Ssp4-HA fusion at the normal chromosomal location, it was found that the level of cellular Ssp4 was decreased in the Δ*tssE*Δ*hcp1* mutant compared with the single Δ*tssE* mutant, confirming that Ssp4 is indeed an Hcp1-stabilised effector (Fig 3C).

One way to explain the decreased killing ability of a *ΔvgrG2* strain is that in the absence of VgrG2, there is simply insufficient VgrG available to form the normal number of active T6SS machineries. To determine if this is the case, additional VgrG1 was expressed from a plasmid in the *ΔvgrG2* and *ΔvgrG1ΔvgrG2* strains. Expression of VgrG1 *in trans* in the double *ΔvgrG1ΔvgrG2* mutant was able to fully restore anti-bacterial activity back to the level of the single *ΔvgrG2* mutant against *P*. *fluorescens* (Fig 3D) and, similarly, to restore delivery of Ssp4 against the non-immune target strain (Fig 3E). However overexpression of VgrG1 could not compensate for the loss of VgrG2, with no restoration of activity to the single *ΔvgrG2* mutant. Additionally, the data indirectly suggest that VgrG proteins compete for limited T6SS complexes and thus VgrG availability is not a limiting factor. In the Δ*vgrG1* mutant, a clear increase in efficiency of Ssp2-mediated anti-bacterial activity and deployment of Rhs1 was observed (Figs 3A and 2A; difference between Δ*vgrG1* mutant and wild type in each case having p < 0.05), and similarly killing of an *E*. *coli* target was increased to the point where no recovered target was detected (S1B Fig). These results imply that loss of competition with VgrG1 for incorporation into the T6SS had allowed an increase in the more efficient VgrG2-mediated activity. Together, these data imply that VgrG2 has a specific role in delivery of even Hcp-dependent cargo effectors and that the T6SS machinery displays a more efficient ability to attack target cells when using VgrG2 than VgrG1.

### Analysis of VgrG specificity by a secretomic approach

**Fig 4:**
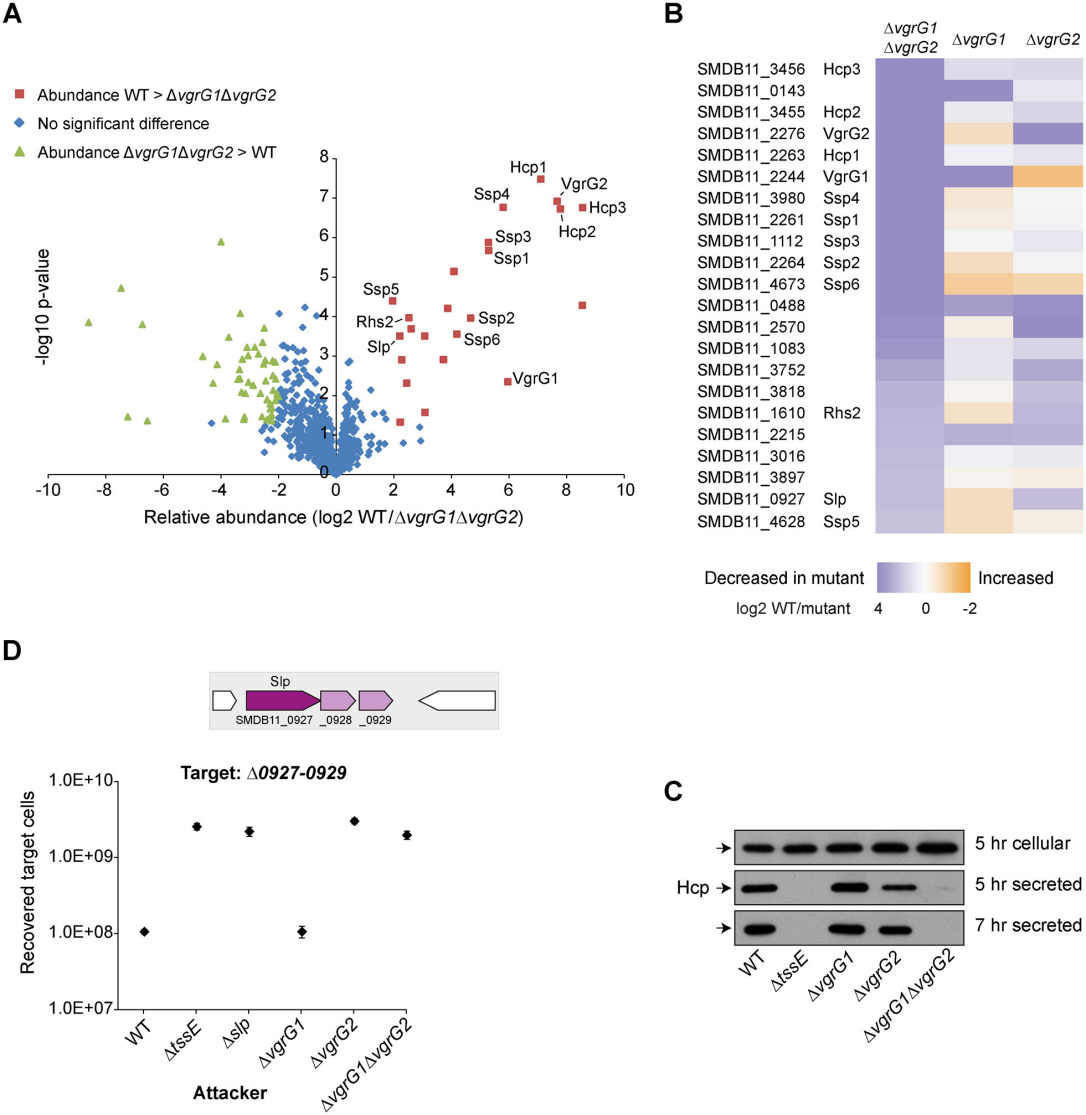
Quantitative secretomics allows comparison of individual VgrG dependence across multiple T6SS substrates and reveals a new VgrG2-specific effector. (A) Volcano plot summarising the secretomic comparison between the wild type (WT) and the T6SS-inactive double VgrG mutant (Δ*vgrG1*Δ*vgrG2*) strains of *S*. *marcescens* Db10. The log2 of the ratios of peptide intensities between the wild type and the Δ*vgrG1*Δ*vgrG2* mutant are plotted against the p-values for label-free quantitation data intensities. Red squares correspond to proteins significantly more abundant in the wild type than the Δ*vgrG1*Δ*vgrG2* mutant (WT/Δ*vgrG1*Δ*vgrG2* > 4; p < 0.05), green triangles to those significantly more abundant in the mutant (WT/Δ*vgrG1*Δ*vgrG2* < 0.25; p < 0.05) and blue diamonds to those without significant changes. (B) Heat map illustrating individual VgrG dependences for each of the proteins significantly decreased in abundance in the secretome of the Δ*vgrG1*Δ*vgrG2* mutant (criteria above and significant ANOVA score overall). Note that proteins significantly increased in the double mutant are not included. (C) Immunoblot detection of secreted Hcp1 after 5 h or 7 h growth for wild type or mutant (Δ*tssE*, Δ*vgrG1*, Δ*vgrG2* and Δ*vgrG1*Δ*vgrG2*) strains of *S*. *marcescens* Db10. (D) Recovery of a target strain lacking *SMDB11_0927–0929* (*S*. *marcescens* Db10 Δ*0927–0929*), following co-culture with wild type or mutant (Δ*tssE*, Δ*slp*, Δ*vgrG1*, Δ*vgrG2* and Δ*vgrG1*Δ*vgrG2*) strains of Db10 as attacker. Points show mean ± SEM (n = 4). Inset: genetic arrangement of *SMDB11_0927–0929*, where *SMDB11_0927* encodes the new lipase-like effector, Slp.

The results above indicate that different effectors can show differing VgrG dependencies, as observed by the impact of loss of specific VgrG proteins on their delivery into target cells. To gain a more complete picture, we decided to measure secretion of all the effectors in *S*. *marcescens* Db10 simultaneously using a proteomic approach. In this experiment, the relative level of protein in the extracellular medium is determined, reporting on the amount successfully ‘fired’ from the secreting cell by the T6SS in the presence and absence of specific VgrG proteins. Total secreted protein from the wild type, Δ*vgrG1*, Δ*vgrG2* and Δ*vgrG1*Δ*vgrG2* mutants was analysed by label-free quantitative mass spectrometry, as we have described previously [42]. We quantified a total of 1025 proteins at a false-discovery rate (FDR) <1% (Fig 4A, S1 Dataset, S3 Fig). Initially, we identified the T6SS-dependent secreted proteins, on the basis of their abundance in the secretome of the wild type being > 4-fold greater than in the secretome of the Δ*vgrG1*Δ*vgrG2* mutant, whose T6SS is inactive (Table 1). Within these 22 proteins, we observed VgrG1 and VgrG2, all three Hcp homologues, and the small secreted effectors Ssp1-Ssp6 as previously [42]. Two other proteins observed for the first time to show T6SS-dependence by this approach were Rhs2 (shown genetically to be a T6SS effector [18]) and SMDB11_0927. SMDB11_0927 is encoded by a gene adjacent to a small gene of unknown function, making it a good candidate for a new effector with associated immunity protein. Most of the other hits have well-known cellular functions and/or have no candidate associated immunity proteins and their connection, if any, with the T6SS is currently unclear. Additionally, a number of cellular proteins were significantly upregulated in the secretome of the Δ*vgrG1*Δ*vgrG2* mutant (Fig 4A, S1 Dataset), in contrast with our equivalent previous secretomic study, when no proteins were significantly upregulated in a T6SS-inactive Δ*tssH* mutant [42]. These upregulated proteins include several stress response proteins (e.g. CspC) and their increased relative levels in the secretome suggest an altered abundance inside the cell, perhaps indicating that incomplete T6SSs containing partially-assembled baseplates assemble in a Δ*vgrG1*Δ*vgrG2* mutant and that this has a deleterious impact on the cell.

**Table 1:** VgrG-dependent secreted proteins in *Serratia marcescens* analysed using label-free mass spectrometry.

Identifier	Name	Description	WT / Δ*vgrG1*Δ*vgrG2*		WT / Δ*vgrG1*		WT / Δ*vgrG2*		ANOVA	Unique peptides	Coverage (%)	MW (kDa)
			log2 ratio	t-test	log2 ratio	t-test	log2 ratio	t-test				
SMDB11_3456	Hcp3	Hcp (TssD) homologue	8.6	+	1.0		1.2	+	+	12	88	17.4
SMDB11_0143		MutS mismatch repair protein	8.5	+	8.5	+	0.6		+	4	6	94.9
SMDB11_3455	Hcp2	Hcp (TssD) homologue	7.8	+	0.5		1.3	+	+	8	59	17.2
SMDB11_2276	VgrG2	VgrG (TssI) homologue	7.7	+	-0.6		7.6	+	+	23	51	71.3
SMDB11_2263	Hcp1	Hcp (TssD) homologue	7.1	+	0.3		0.8	+	+	22	100	16.8
SMDB11_2244	VgrG1	VgrG (TssI) homologue	6.0	+	7.3	+	-1.3	+	+	29	48	87.5
SMDB11_3980	Ssp4	T6SS effector	5.8	+	-0.4		0.1		+	7	25	35.5
SMDB11_2261	Ssp1	T6SS amidase effector (Tae4.1^SM^)	5.3	+	-0.2		0.2		+	6	42	18.2
SMDB11_1112	Ssp3	T6SS effector	5.3	+	0.1		0.6	+	+	7	37	20.6
SMDB11_2264	Ssp2	T6SS amidase effector (Tae4.2^SM^)	4.7	+	-0.6		0.2		+	11	77	17.9
SMDB11_4673	Ssp6	T6SS effector	4.2	+	-1.0		-0.8	+	+	6	34	24.5
SMDB11_0488		KdpD sensor histidine kinase	4.1	+	3.4	+	3.9	+	+	3	3	99.7
SMDB11_2570		Putative exported protein	3.9	+	-0.2		4.1	+	+	2	25	11.3
SMDB11_1083		Unknown function	3.7	+	0.8		1.3		+	5	34	25.7
SMDB11_3752		AceA isocitrate lyase	3.1	+	0.7		3.0	+	+	6	18	47.3
SMDB11_3818		TauD taurine dioxygenase	2.6	+	-0.1		2.1		+	4	19	32.3
SMDB11_1610	Rhs2	T6SS-associated Rhs protein	2.5	+	-0.5		2.4	+	+	6	6	158.0
SMDB11_2215		FlgF flagellar rod protein	2.4	+	2.7	+	2.6	+	+	2	13	26.5
SMDB11_3016		IscA Fe-S cluster assembly protein	2.3	+	0.3		0.5		+	2	23	11.6
SMDB11_3897		Putative metal-binding protein	2.2	+	-0.1		-0.2		+	5	35	26.1
SMDB11_0927	Slp	Putative (phospho)lipase	2.2	+	-0.6		2.2	+	+	6	14	60.3
SMDB11_4628	Ssp5	T6SS effector	2.0	+	-0.6	+	-0.2		+	6	23	29.4

Proteins are included on the basis of their abundance in the secretome being > 4-fold greater in the wild type than in the double Δ*vgrG1*Δ*vgrG2* mutant (i.e. being T6SS-dependent), with significant t-test (p<0.05, +) for this comparison and significant ANOVA score (p<0.05, +) over the four conditions. The table summarises the data from four independent biological replicates of wild type (WT) *S*. *marcescens* Db10 and the single and double *vgrG* mutants, analysed using label-free quantitative mass spectrometry. No peptides from VgrG1 and VgrG2 were detected in their respective mutants, the ratio was determined by imputing correlating intensities around the detection limit. Note that Ssp5 showed a 3.9-fold ratio but since it is a known effector and the only other significantly increased protein in the dataset, it is included.

Within the group defined as T6SS-dependent secreted proteins, we then considered dependence on individual VgrG homologues, graphically depicted in Fig 4B. Several proteins showed total dependence on VgrG2, with the same decrease in abundance in the Δ*vgrG2* mutant as in the Δ*vgrG1*Δ*vgrG2* mutant (but not in Δ*vgrG1*). These included Rhs2, confirming the genetic result in Fig 2B, and the candidate effector SMDB11_0927. One protein, MutS (SMDB11_0143), showed strong VgrG1 dependence, but this is likely to be an indirect effect rather than MutS itself being a T6SS-secreted effector (see Discussion). Rhs1 (SMDB11_2278) was not identified as a T6SS-dependent secreted protein in this analysis, because its abundance was too low for robust detection in the wild type samples. However it was significantly increased (4.2-fold, p<0.05) in the secretome of the Δ*vgrG1* mutant (S1 Dataset), indirectly implying dependence on VgrG2 in support of the genetic data (Fig 2A). The results in Fig 3 imply that some effectors, including Ssp2 and Ssp4, whilst not depending totally on VgrG2, do show a preference for it. However examination of the proteomic data indicates that levels of secreted Ssp2 and Ssp4 show no significant difference between the wild type and the Δ*vgrG2* mutant (Table 1). Similarly, Ssp1, Ssp3, Ssp5 and Ssp6 do not show a significant reduction in secretion in the Δ*vgrG2* mutant (considering significant to be > 1.5-fold changed in abundance in the Δ*vgrG2* but not the Δ*vgrG1* mutant, p<0.05). It is interesting to note that two of the three Hcp homologues also appear to show a modest preference for VgrG2, Hcp1 (1.75-fold) and Hcp2 (2.5-fold), compared with 140- or 380-fold changes relative to the Δ*vgrG1*Δ*vgrG2* mutant. Consistent with this, if Hcp1 secretion is measured by immunoblot at an earlier timepoint than in our standard assay, a small decrease can be detected in the Δ*vgrG2* but not the Δ*vgrG1* mutant (Fig 4C), indicating that it is possible to detect a minor preference for VgrG2 in basic T6SS activity under certain conditions. Whether this is sufficient to account for the larger impact observed in the competition setting remains to be proven.

### Identification of a new VgrG2-dependent cargo T6SS effector

The secretome analysis identified the SMDB11_0927 protein as a strong candidate for a new T6SS- and VgrG2-dependent secreted effector. Indeed re-examination of the data from our previous secretome experiment [42] revealed that this protein was also significantly reduced in abundance in the secretome of a TssH mutant compared with the wild type (p<0.05), however on that occasion it just missed the chosen 4-fold threshold. SMDB11_0927 is encoded outside the main T6SS gene cluster in a three-gene locus with two downstream open reading frames encoding homologous small proteins that could potentially represent cognate immunity proteins (Fig 4D, inset). Furthermore, SMDB11_0927 contains a conserved GxSxG motif found in several families of phospholipase effectors associated with T6SSs [22] and was renamed Slp (Secreted lipase-like protein). Mutants of *S*. *marcescens* Db10 with in-frame deletions of *SMDB11_0927* and *SMDB11_0927–0929* were constructed, and susceptibility of the Δ*SMDB11_0927–0929* strain (which lacks both putative immunity proteins) to T6SS mediated anti-bacterial activity was assessed (Fig 4D). Co-culture of this target strain with a wild type attacker resulted in >10x reduction in recovered viable target cells compared with when the target was co-cultured with a T6SS mutant (Δ*tssE*). Importantly, this anti-bacterial activity was dependent on the presence of the putative toxin Slp (SMDB11_0927) in the attacker, being eliminated in the Δ*slp* mutant and therefore confirming that Slp is a new T6SS-delivered antibacterial effector. Slp-dependent killing was also abolished when the target strain Δ*SMDB11_0927–0929* was co-cultured with a Δ*vgrG2* but not a Δ*vgrG1* mutant attacker, confirming its VgrG2-dependency (Fig 4D). Additionally, confirming that the loss of antibacterial activity in the Slp mutant was not due to a loss of T6SS functionality, Hcp1 and effector secretion was unaffected (S4A Fig). The phenotype of the Δ*slp* mutant could also be fully complemented by the expression of Slp *in trans* (S4B Fig).

### A functional T6SS can utilise distinct VgrG-PAAR based assemblies in *S*. *marcescens*

**Fig 5:**
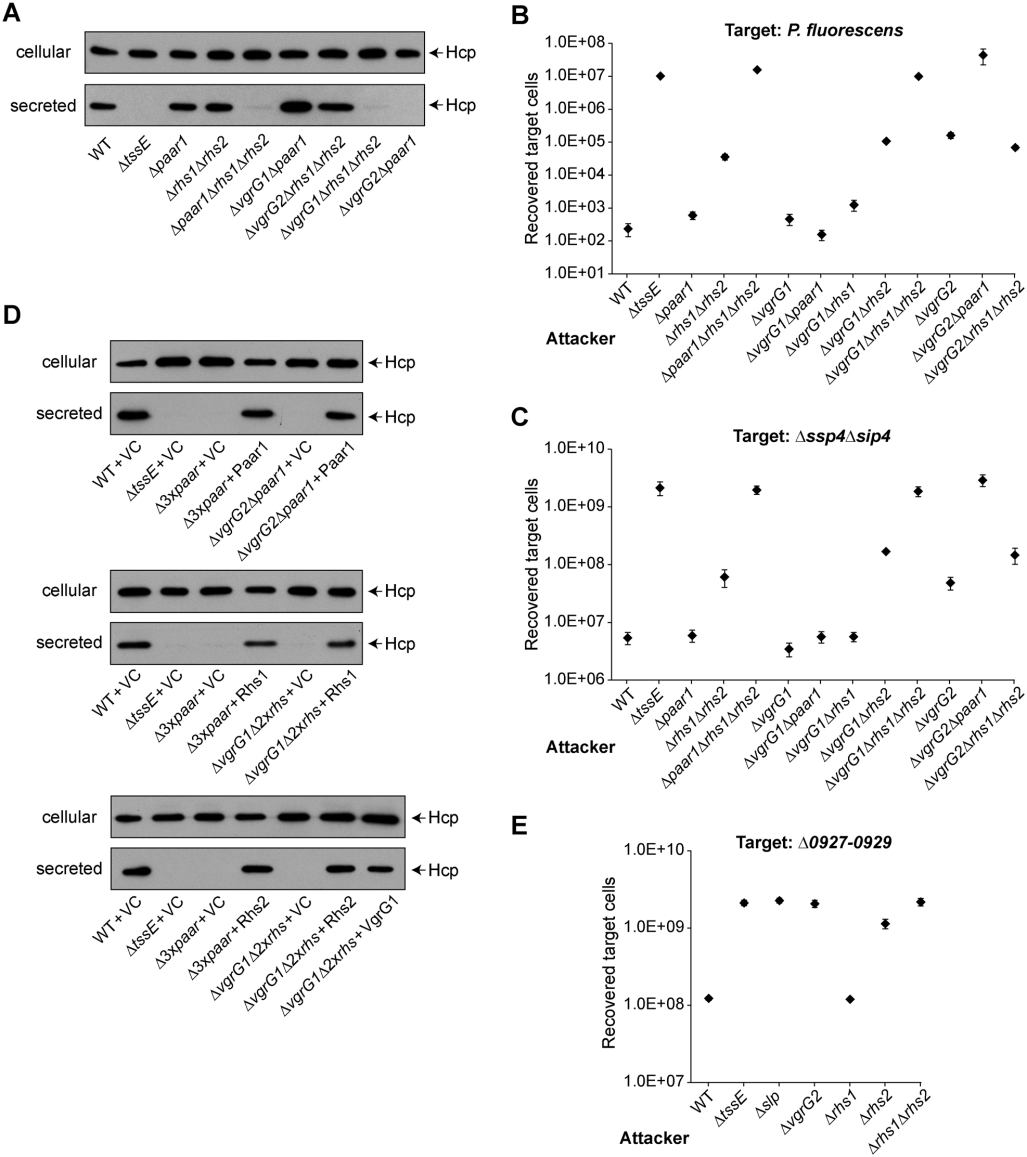
VgrG1 and VgrG2 require specific PAAR proteins to assemble a functional T6SS. (A) Immunoblot detection of Hcp1 in cellular and secreted fractions of wild type (WT) or mutant (Δ*tssE*, Δ*paar1*, Δ*rhs1*Δ*rhs2*, Δ*paar*Δ*rhs1*Δ*rhs2*, Δ*vgrG1*Δ*paar1*, Δ*vgrG2*Δ*rhs1*Δ*rhs2*, Δ*vgrG1*Δ*rhs1*Δ*rhs2* and Δ*vgrG2*Δ*paar1*) strains of *S*. *marcescens* Db10. (B-C) Recovery of target organisms *P*. *fluorescens* 55, part B, or *S*. *marcescens* Db10 Δ*ssp4*Δ*sip4*, part C, following co-culture with wild type or mutant strains of Db10 as attacker (mutants as part A, with also Δ*vgrG1*, Δ*vgrG2*, Δ*vgrG1*Δ*rhs1* and Δ*vgrG1*Δ*rhs2*). Points show mean +/- SEM (n = 4). (D) Hcp1 secretion by wild type or mutant strains carrying either the vector control plasmid (+VC) or plasmids directing expression of Paar1 (+Paar1, pSC734), Rhs1 and RhsI1 (+Rhs1, pSC791), Rhs2 and RhsI2 (+Rhs2, pSC788), or VgrG1 (+VgrG1, pSC622) *in trans*. The empty vector control was pSUPROM for Paar1, Rhs2 and VgrG1; for Rhs1 the empty vector was pBAD18-Kn and expression was induced with 0.0002% l-arabinose. Abbreviations: Δ3x*paar*, Δ*paar*Δ*rhs1*Δ*rhs2*; Δ2x*rhs*, Δ*rhs1*Δ*rhs2* mutant (E) Recovery of target strain *S*. *marcescens* Db10 Δ*0927–0929* following co-culture with wild type (WT) or mutant (Δ*tssE*, Δ*slp*, Δ*vgrG2*, Δ*rhs1*, Δ*rhs2* and Δ*rhs1*Δ*rhs2*) strains of Db10.

It has been shown that PAAR/PAAR-containing proteins, which are able to sit on the tip of the VgrG trimer, can be very important, perhaps even essential, components of the T6SS [28]. However we found previously that deletion of both Rhs1 and Rhs2 does not result in an inactive T6SS [18]. Examination of the T6SS cluster of *S*. *marcescens* Db10 identified a small PAAR protein, in addition to Rhs1 and Rhs2. This protein, which we name Paar1, is encoded near to *vgrG1* by *SMDB11_2250* (Fig 1A). To define the role and requirement of Paar1, Rhs1 and Rhs2 in the T6SS of *S*. *marcescens*, deletion mutants Δ*paar1*, Δ*rhs1*Δ*rhs2* and Δ*paar1*Δ*rhs1*Δ*rhs2* were constructed and the functionality of the T6SS secretion determined by monitoring Hcp1 secretion and anti-bacterial activity (Fig 5A–5C). Deletion of *paar1* did not affect T6SS-dependent secretion or anti-bacterial activity against *P*. *fluorescens* and an Ssp4-susceptible target strain, similar to loss of VgrG1. Deletion of *rhs1rhs2* did not impair secretion to the medium, but did cause a reduction in the efficiency of T6SS-dependent anti-bacterial activity against both targets, similar to the loss of VgrG2. In contrast, simultaneous inactivation of all three PAAR proteins (Δ*paar1*Δ*rhs1*Δ*rhs2*) abolished T6SS function, with a loss of secretion and no anti-bacterial activity against any target strain.

Given the phenotypes of these *paar* mutants and the finding that both Rhs1 and Rhs2 depend on VgrG2, we hypothesised that Paar1 is a VgrG1-specific PAAR protein and that the *S*. *marcescens* T6SS can form two distinct functional assemblies. If this is true, then deletion of the PAAR(s) from one assembly and the VgrG from the other would inactivate both versions and result in an inactive T6SS machinery. Hereafter, for simplicity, we will refer to VgrG1-Paar1 and VgrG2-Rhs1/Rhs2 as “Assembly 1” and “Assembly 2”, respectively. To test this idea, deletion mutants lacking *vgrG1* in combination with deletion of *paar1*, *rhs1*, *rhs2* or *rhs1rhs2*, and lacking *vgrG2* in combination with deletion of *paar1* or *rhs1rhs2*, were constructed and their T6SS functionality monitored as above (Fig 5A–5C). Confirming our hypothesis, simultaneous loss of VgrG and PAAR(s) from the same assembly (Δ*vgrG1*Δ*paar1* or Δ*vgrG2*Δ*rhs1*Δ*rhs2*) did not affect secretion or anti-bacterial activity, compared with deletion of either VgrG or PAAR(s) alone. In contrast, loss of VgrG1 in combination with Rhs1Rhs2, or loss of VgrG2 in combination with Paar1, completely abrogated T6SS activity. Of note, the phenotypes of the *paar* mutants could be complemented *in trans*. Re-introduction of *paar1*, *rhs1* or *rhs2* into the triple Δ*paar1*Δ*rhs1*Δ*rhs2* was able to fully restore Hcp1 secretion. Similarly, introduction of *paar1* in the Δ*vgrG2*Δ*paar1* mutant, or introduction of *rhs1*, *rhs2* or *vgrG1* in the Δ*vgrG1*Δ*rhs1*Δ*rhs2* mutant, restoring a complete assembly in each case, fully restored secretion *in vitro* (Fig 5D). Finally, as also predicted by our model, Paar1 is not required for delivery of Rhs1, Rhs2 or Slp (S5 Fig).

Reinforcing the observation that one functional combination of VgrG and PAAR protein is necessary and sufficient for T6SS activity, the Δ*vgrG1*Δ*rhs1* and Δ*vgrG1*Δ*rhs2* mutants (with intact VgrG2-Rhs2 or VgrG2-Rhs1 assemblies, respectively) still displayed anti-bacterial activity, even if not necessarily at wild type levels (Fig 5B and 5C). However, it is clear that the two combinations are not equally efficient. Considering activity against the Δ*sip4* target strain, where the toxic effector domains of each Rhs protein will have no impact since the target encodes the cognate immunity proteins, it is apparent that VgrG2-Rhs2 alone (Δ*vgrG1*Δ*rhs1*) functions as well as the wild type, whereas VgrG2-Rhs1 alone (Δ*vgrG1*Δ*rhs2*) is considerably less efficient (Fig 5C). Similarly, VgrG2-Rhs1 is less efficient than VgrG2-Rhs2 against *P*. *fluorescens* (Fig 5B). Therefore we decided to further divide Assembly 2 into “Assembly 2.1” (VgrG2-Rhs1) and “Assembly 2.2” (VgrG2-Rhs2). Given that Slp is a VgrG2- and therefore Assembly 2-specific effector, we next asked whether Slp would also display any preference for Assembly 2.1 or 2.2. This revealed that a mutant lacking Rhs2 displayed a strong reduction in Slp-mediated growth inhibition, although remaining able to deliver the effector (Fig 5E; difference between Δ*rhs2* and Δ*slp* is significant, p < 0.01). In contrast, an Rhs1 mutant could deliver Slp as well as the wild type. Therefore both an VgrG2-specific and an Hcp1-dependent cargo effector are delivered more efficiently by Assembly 2.2 than Assembly 2.1.

### VgrG2 cannot be deployed in the absence of Rhs1 and Rhs2

**Fig 6:**
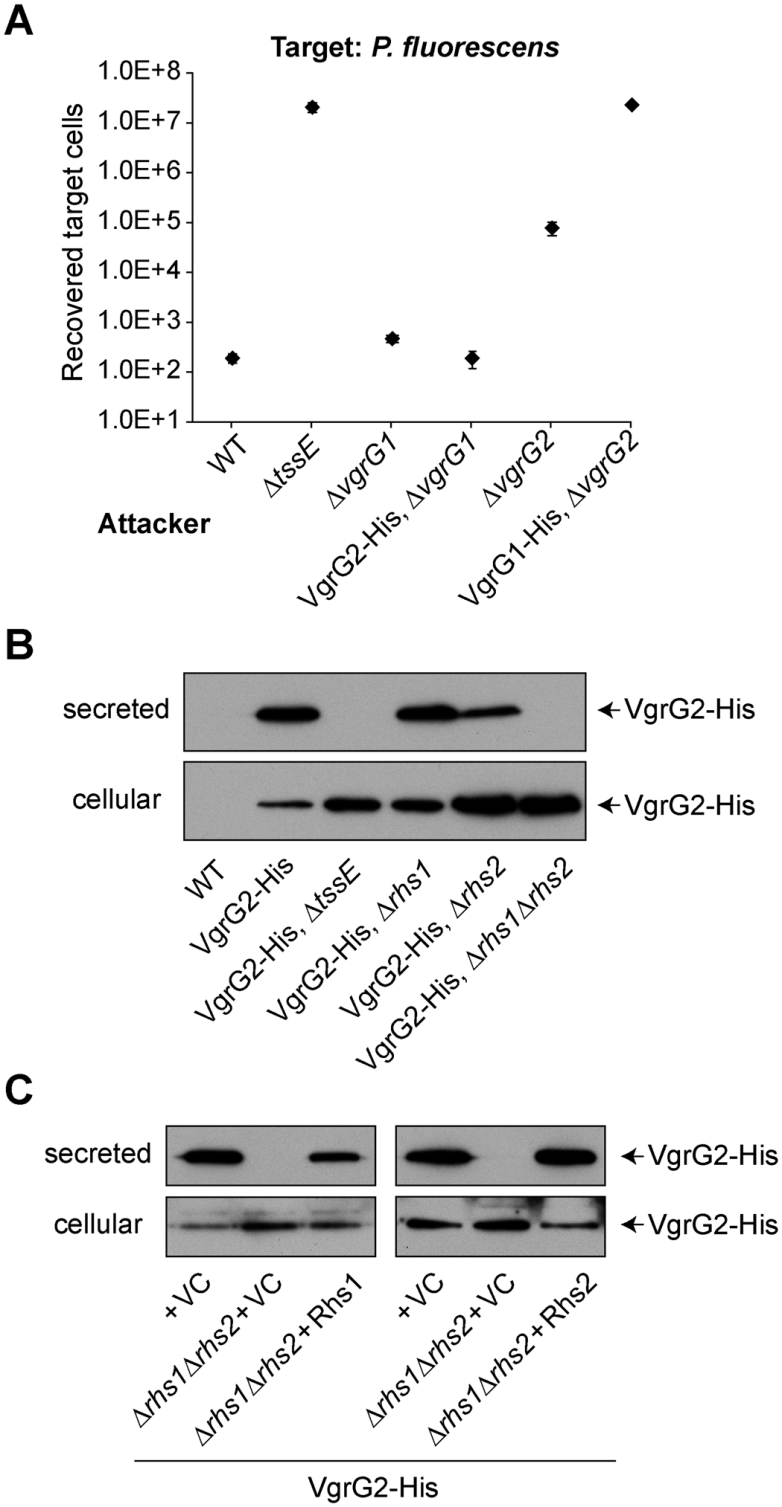
VgrG2 requires at least one of its specific PAAR proteins for secretion but not stability. (A) A VgrG2-His_6_ fusion protein (VgrG2-His) encoded at the normal chromosomal location is fully functional, whereas VgrG1-His_6_ (VgrG1-His) is non-functional. Recovery of target organism *P*. *fluorescens* 55 following co-culture with wild type *S*. *marcescens* Db10 (WT), the Δ*tssE*, Δ*vgrG1* and Δ*vgrG2* mutants, or strains expressing VgrG2-His in the Δ*vgrG1* background or VgrG1-His in the Δ*vgrG2* background. Points show mean +/-SEM (n = 4). (B) Anti-His immunoblot of cellular and secreted protein from wild type *S*. *marcescens* Db10 or strains expressing the chromosomal VgrG2-His_6_ fusion protein, either in a wild type background (VgrG2-His) or in strains lacking TssE (VgrG2-His, Δ*tssE*), Rhs1 (VgrG2-His, Δ*rhs1*), Rhs2 (VgrG2-His, Δ*rhs2*) or both Rhs proteins (VgrG2-His, Δ*rhs1*Δ*rhs2*). (C) Immunoblot detection of VgrG2-His in the cellular and secreted fractions of strains expressing VgrG2-His in a wild type or Δ*rhs1*Δ*rhs2* background and carrying either the vector control plasmid (+VC) or plasmids directing the expression of Rhs1 and RhsI1 (+Rhs1, pSC791) or Rhs2 and RhsI2 (+Rhs2, pSC788) *in trans*. The empty vector control for Rhs2 was pSUPROM; for Rhs1 it was pBAD18-Kn and expression was induced with 0.0002% l-arabinose.

Having defined specific, functional VgrG-PAAR combinations genetically, we considered whether VgrG proteins can still be secreted from the cell in the absence of their cognate PAAR protein(s). This might be possible, for example, within a heterotrimer with another VgrG homologue. To address this question, a gene encoding a C-terminal hexahistidine-tagged version of *vgrG2* (VgrG2-His) was used to replace the native *vgrG2* gene on the chromosome of the wild type, Δ*rhs1*, Δ*rhs2* and Δ*rhs1*Δ*rhs2* strains. The VgrG2-His fusion protein was shown to be fully functional by its ability to kill *P*. *fluorescens* and secrete substrates at wild type levels in a Δ*vgrG1* background (Fig 6A and S4 Fig). In contrast, the equivalent VgrG1-His fusion protein was found to be non-functional (Fig 6A), as were several other VgrG1 fusion proteins tested. Cellular and secreted levels of VgrG2-His were monitored by immunoblotting (Fig 6B). As expected, loss of one Rhs did not inhibit use and, therefore, secretion of VgrG2. However, simultaneous deletion of both Rhs proteins resulted in complete loss of VgrG2-His secretion, suggesting that a VgrG cannot be used as part of the T6SS machinery when its specific PAAR is not present. Importantly, intracellular VgrG2-His levels did not decrease when both Rhs1 and Rhs2 were deleted, demonstrating that VgrG stability does not depend on the PAAR protein (Fig 6B). Secretion of VgrG2-His could be restored in the Δ*rhs1*Δ*rhs2* background by expression of either Rhs1 or Rhs2 *in trans* (Fig 6C).

### Isolation of native tip complexes containing VgrG2, Rhs and EagR proteins

**Fig 7:**
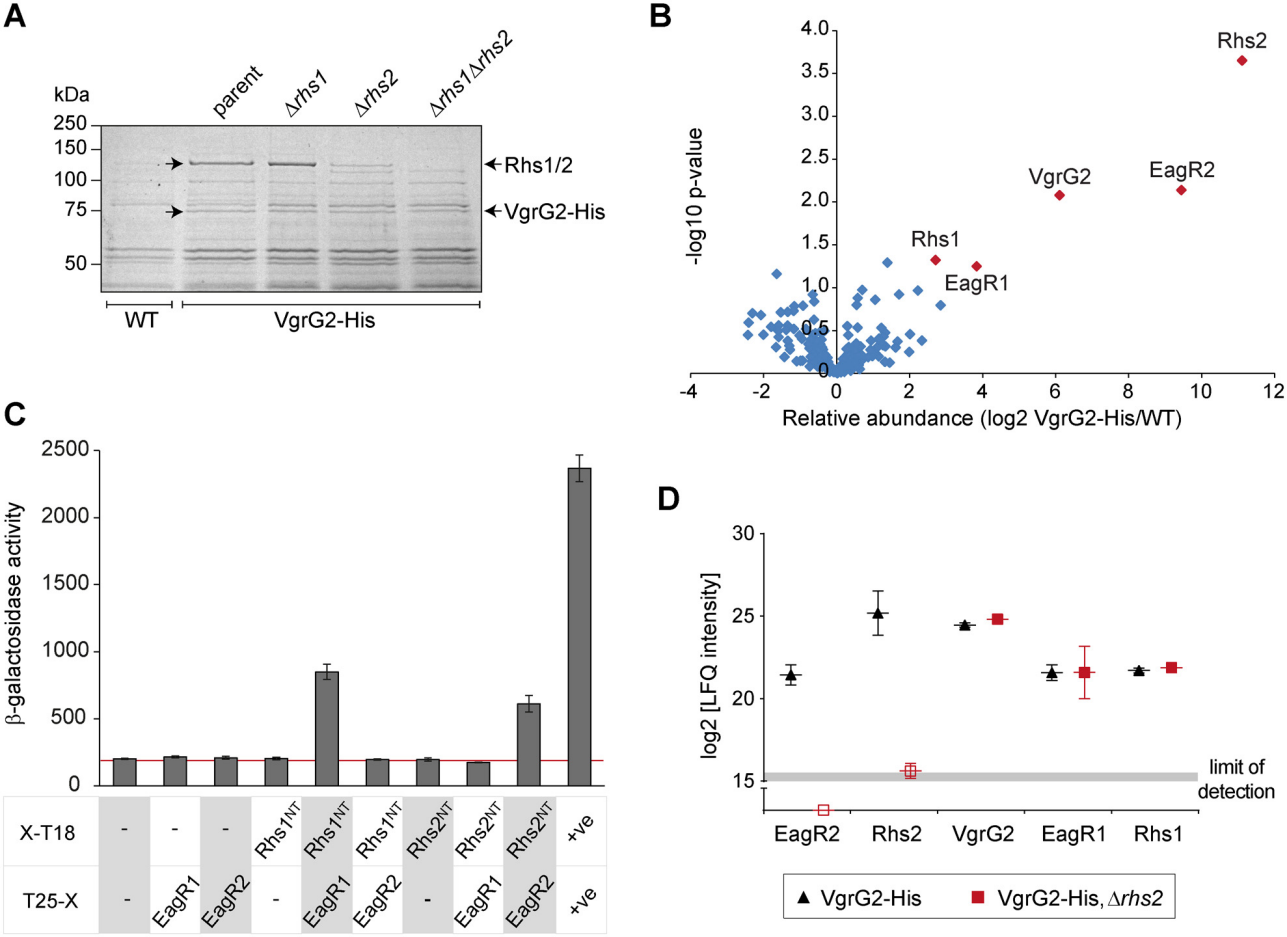
Isolation of native VgrG and PAAR containing complexes. (A) Identification of VgrG2-associated proteins by small-scale affinity purification. Total cellular proteins from *S*. *marcescens* Db10 encoding the VgrG2-His_6_ fusion protein, in an otherwise wild type genetic background (parental) or in strains lacking Rhs1 (Δ*rhs1*) or Rhs2 (Δ*rhs2*) or both Rhs proteins (Δ*rhs1*Δ*rhs2*), or from the wild type (WT, no His_6_ tag) as a negative control, were incubated with Ni^2+^ beads to co-isolate VgrG2-His_6_ (VgrG2-His) with any bound proteins. Eluted proteins were resolved by SDS-PAGE and visualised by Coomassie staining. Arrows indicate the positions of two VgrG2-His specific bands, one corresponding to VgrG2-His (predicted MW 72 kDa) and one corresponding to a mixture, in the parental strain, of Rhs1 and Rhs2 (predicted MW 165 and 158 kDa, respectively); identifications were confirmed by mass spectrometry. (B) Quantitative analysis of the proteins present in samples isolated as above by affinity purification from the control strain (WT) or the strain carrying the VgrG2-His fusion (in an otherwise wild type background) by solution mass spectrometry. Proteins were identified as being associated with VgrG2 when significantly enriched in the VgrG2-His sample compared with the WT sample, with relative abundance in VgrG2-His/WT > 4-fold, p<0.05 (red diamonds) (C) Bacterial two-hybrid assay to detect interactions between the N-terminal domain of Rhs1 or Rhs2, each fused with T18 (Rhs1^NT^-T18, pSC699, and Rhs2 ^NT^ -T18, pSC700), and full-length EagR1 or EagR2, each fused with T25 (T25-EagR1, pSC688, and T25-EagR2, pSC689). Negative controls were provided by the empty vectors, pUT18 and pT25 (-), and a positive control by the self-interaction of TssK (+ve; TssK-T18, pSC048, and T25-TssK, pSC053). Shown is β-galactosidase activity, expressed as Δ405/min/ml/OD_600_, of the reporter strain transformed with the combinations of plasmids indicated. Bars show mean +/- SEM (n = 3 independent transformations) and the background level of activity is indicated by a red line. (D) As for part B, except that the VgrG2-His affinity purification was performed in the parental and Δ*rhs2* backgrounds. The abundance of each of the VgrG2-associated proteins identified in part B in the two strains is given (represented as the log2 of the label-free quantitation intensities), with the effective limit of detection indicated and points showing mean +/- SD (n = 3).

Having shown that VgrG2 is essential for delivery of Rhs1 and Rhs2 and also depends on them for its own secretion, we decided to investigate physical interactions between these proteins by affinity isolation of native VgrG2-containing complexes using the chromosomally-encoded VgrG2-His protein described above. Upon isolation of VgrG2, a specific co-purifying band was observed with apparent MW between 100 and 150 kDa which was found to contain Rhs1 and Rhs2, which both have a predicted MW of ~160 kDa. Consistently, this band was observed to disappear in a strain lacking both Rhs proteins as well as significantly reduce in intensity in an Rhs2 mutant (Fig 7A). Further, quantitative, confirmation of this result was performed by label-free quantitative mass spectrometry analysis of the total eluate from the isolation of VgrG2-His from an otherwise wild type strain, compared with a control lacking the fusion protein (Fig 7B). This analysis identified five proteins significantly over-represented in the VgrG2-His eluate compared with control, namely VgrG2, Rhs1, Rhs2, EagR1 and EagR2. Eag is a recently described class of effector-associated factors involved in delivery of some T6SS effectors and we have previously shown that EagR1 is specifically required for delivery of Rhs1 [18, 36]. Since a single PAAR protein fully occupies the tip of a VgrG trimer [28], the two Rhs proteins cannot simultaneously occupy the same VgrG structure. Thus we hypothesised that VgrG2 can interact either with Rhs1 and EagR1 (Assembly 2.1) or with Rhs2 and EagR2 (Assembly 2.2). EagR2 (SMDB11_1609), a homologue of EagR1, is encoded immediately upstream of Rhs2, in the equivalent genetic context to EagR1 (Fig 1A). As predicted, an Δ*eagR2* mutant was found to be specifically required for delivery of Rhs2, being required for killing of a Δ*rhsI2* but not Δ*rhsI1* target strain, the converse of the Δ*eagR1* mutant (S6 Fig).

To prove a direct interaction between Rhs1 and EagR1 and between Rhs2 and EagR2, we utilised the bacterial two-hybrid system [43]. We hypothesized that EagR proteins should interact with the conserved, PAAR-containing N-terminal domains of Rhs proteins (which we defined as amino acids 1–422 for Rhs1^NT^ and 1–363 for Rhs2^NT^). By fusion of Rhs^NT^ to the N-terminus of T18 and EagR to the C-terminus of T25, specific interactions between Rhs1^NT^ and EagR1 and between Rhs2^NT^ and EagR2 (but not vice versa) were observed (Fig 7C). By further utilising all possible combinations of Rhs1^NT^ and EagR1, we were able to demonstrate both the reciprocal interaction (EagR1-T18 with T25-Rhs1^NT^) and a strong self-interaction between EagR1-T18 and T25-EagR1 (S6A Fig). Self-interaction of EagR2 was also observed, consistent with a proposed dimeric organisation for Eag-family proteins [36].

We were further interested in understanding how EagR and Rhs proteins depend on each other for interaction with VgrG. It has been suggested that EagR-like proteins are required for interactions between VgrG and PAAR domain-containing effectors at least partly because the PAAR protein becomes unstable in their absence [36]. Consistent with this, Rhs2 was no longer co-purified when the original VgrG2-His affinity purification was performed in a Δ*rhs1*Δ*eagR2* background (S7A Fig), and an epitope tagged version of Rhs1 appeared to be less stable when expressed *in trans* in a Δ*eagR1* background than in the wild type (S7B Fig). Conversely, to investigate whether EagR proteins interact directly with VgrG proteins or rather depend on the cognate Rhs protein for their association with VgrG, we repeated the VgrG2-His affinity purification in a wild type and a Δ*rhs2* background. Analysis of the eluate by mass spectrometry revealed that whilst Rhs1 and EagR1 co-purified with VgrG2-His in both WT and Δ*rhs2* background, EagR2 was no longer associated with VgrG2-His in the absence of Rhs2 (Fig 7D).

All together, these data demonstrate that the T6SS of *Serratia marcescens* incorporates stable VgrG2-Rhs1-EagR1 and VgrG2-Rhs2-EagR2 complexes, entirely consistent with the Assemblies 2.1 and 2.2 defined initially by a genetic approach.

## Discussion

In this work, we have used genetic and biochemical approaches to uncover new aspects of effector recognition and deployment by the T6SS and discovered that important subtleties exist beyond the current model. This model envisages that certain effectors are absolutely dependent on one specific VgrG whereas others are Hcp-dependent and utilise any VgrG indiscriminately, and that each PAAR protein interacts with a different VgrG protein [30, 31, 34]. In contrast, we reveal that the efficiency of delivery of cargo effectors can be modulated according to the VgrG-PAAR combination used and that two distinct PAAR-containing Rhs proteins can utilise one VgrG. Further, we show that whilst distinct and specific VgrG-PAAR combinations can support T6SS function, at least one PAAR protein is essential for T6SS activity.

Examining the impact of loss of either or both VgrG homologues on the activity of the anti-bacterial T6SS of *S*. *marcescens* Db10, revealed that, as predicted for a core component, the system is inactive if neither VgrG is present. VgrG proteins are close structural homologues of the bacteriophage T4 hub component gp27-gp5, which forms the central part of the baseplate structure prior to sheath contraction [44, 45]. Upon contraction, the baseplate ‘opens’ and the hub is pushed out through the baseplate on the tip of the gp19 tube, equivalent of the proposed expulsion of VgrG at the distal tip of the Hcp tube. Indeed VgrG has recently been confirmed to form part of a T6SS baseplate-like complex [24] and shown to play an essential role in initiating stacked head-to-tail assembly of Hcp hexamers into tubes, which is in turn required for TssBC sheath assembly [46]. Interestingly, however, we discovered that while both VgrG proteins in Db10 were redundant at the level of basic secretion system activity, i.e. the ability to ‘fire’ Hcp and effectors into the medium *in vitro*, loss of VgrG2, but not VgrG1, resulted in a considerable reduction in T6SS-mediated anti-bacterial activity against competitor species. Providing a partial explanation for this observation, we defined three effectors as being specifically VgrG2-dependent: Rhs1, Rhs2 and Slp. Specific VgrG-dependence has been observed for Rhs and phospholipase effectors [30–32, 34, 35] and is consistent with the presence of VgrG-interacting PAAR domains (Rhs) or a larger size precluding Hcp binding (Slp). However, this study represents the first time that two Rhs proteins, or indeed any PAAR proteins, have been found to utilise the same VgrG protein. The existence of multiple functional PAAR pairings for one VgrG increases the potential repertoire of effectors that can be secreted by a given T6SS.

Unexpectedly, we also found that the delivery of cargo effectors which are not strictly dependent on one VgrG can nevertheless show a preference, with both Ssp2 and Ssp4 (expected or shown, respectively, to be Hcp1 dependent cargo effectors) being delivered more efficiently to target cells using VgrG2 than VgrG1. This subtlety has not been reported previously but can make a significant difference to the outcome of an inter-strain competition. In contrast, our proteomic study did not reveal significant differences in the secretion of small cargo effectors, including Ssp2 and Ssp4, to the medium *in vitro*. However a modest decrease in Hcp1 secretion was observed in the VgrG2 mutant, which could be replicated by immunoblot analysis under certain conditions. It is not clear whether this small effect alone, given a cumulative impact over several generations, could be enough to account for the considerable defect in co-culture experiments. Rather there might be another VgrG-related factor contributing to the overall efficiency of T6SS-mediated bacterial killing ‘*in vivo*’, for example a VgrG2-tipped puncturing device being better able to reach, puncture or release effectors in a target cell than one tipped by VgrG1.

Our proteomic analysis allowed us to identify a new T6SS- and VgrG2-dependent anti-bacterial effector in *S*. *marcescens* Db10, Slp, which is predicted to have lipase or phospholipase activity. Such enzymes appear to be common T6SS effectors, comprising at least five broad families, Tle1-5, and normally encoded adjacent to a *vgrG* gene [22]. In EAEC and *V*. *cholerae*, a phospholipase effector has been shown to depend on the corresponding VgrG homologue for delivery and also to interact with it, either directly or using a conserved accessory adaptor protein, respectively [32, 34]. In the latter case, the Tap-1 (or TEC) adaptor protein is encoded immediately upstream of the TseL (Tse2^Vc^) phospholipase [34, 35], the equivalent location of *eag* accessory genes relative to their effector genes [18, 36]. Unusually, Slp is not genetically linked with a *vgrG* gene, but nevertheless it is still specifically dependent on VgrG2 for secretion and delivery to target cells; there is also no obvious candidate for an accessory adaptor protein to mediate interaction of Slp and VgrG2.

VgrG2 is essential for the delivery of at least three effectors and supports efficient T6SS activity alone. But why is VgrG1, and the VgrG1-Paar1 assembly, retained? One possibility is simply as a ‘failsafe’ to ensure an alternative pathway for non-VgrG2-dependent cargo effectors should anything go wrong with the VgrG2-Rhs assembly. Or, the properties of the VgrG1-Paar1 tip itself could be advantageous for certain targets. Another, perhaps more likely, possibility is that other effector(s) specific for VgrG1 remain to be identified, which could interact with either VgrG1 itself or with Paar1. The only protein specifically depleted in the secretome of the VgrG1 single mutant was the MutS mismatch repair protein, which is involved in maintaining genome stability [47]. As a highly conserved protein involved in a critical cellular pathway, MutS is unlikely to be a T6SS-secreted effector. Rather, its decreased presence in the culture supernatant of a Δ*vgrG1* or Δ*vgrG1*Δ*vgrG2* mutant most likely represents an indirect effect on cellular levels of the protein. The reason for this is currently unclear but it might indicate a VgrG1-dependent effector somehow inducing a DNA repair response upon delivery. The two VgrG homologues differ in their C-terminal regions (S1 Fig), which is consistent with this region mediating a different set of interactions between each VgrG and its cognate specific effector, accessory and/or PAAR proteins, and likely explains why addition of a C-terminal His tag inhibits the function of VgrG1 but not VgrG2. It is unclear why overexpression of VgrG2, but not VgrG1, has a dominant negative impact on T6SS activity, but significant loss of stoichiometry with other baseplate components might perhaps cause an accumulation of ‘dead-end’ misassembled complexes.

**Fig 8:**
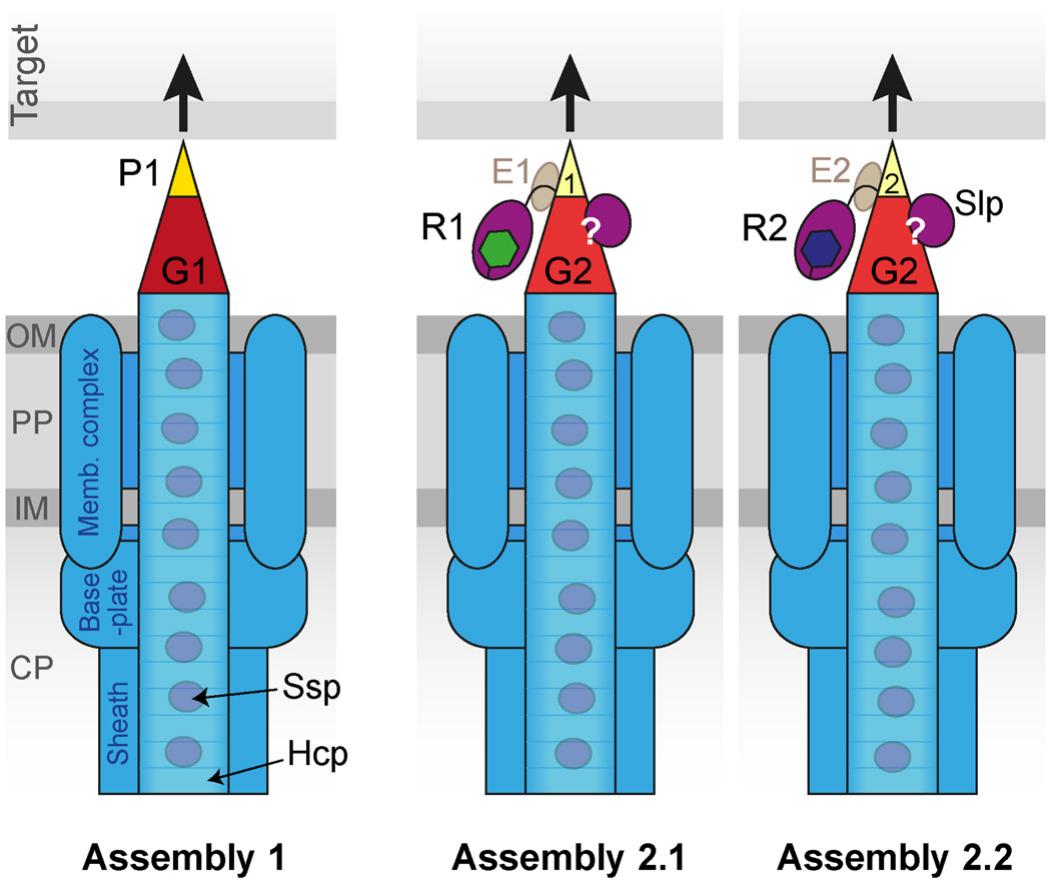
Schematic representation of the three distinct assemblies formed by the T6SS of *S*. *marcescens*. Assembly 1 utilises VgrG1 (G1) and a small, dedicated PAAR protein (Paar1, P1). Assemblies 2.1 and 2.2 both include VgrG2 (G2) together with either Rhs1 (R1; Assembly 2.1) or Rhs2 (R2, Assembly 2.2), with the Rhs proteins supplying their N-terminal PAAR-containing domains (yellow), each bound by the cognate EagR protein (E1 or E2). The VgrG2-specific effector Slp can be delivered by either 2.1 or 2.2 but it is not yet clear whether this is through a direct interaction between Slp and VgrG2 or via an intermediary protein (indicated by ‘?’). If any of the three assemblies are intact, the system can fire and Hcp-dependent cargo effectors can be delivered. However delivery of some, if not all, of these cargo effectors is more efficient using VgrG2, and delivery of at least Ssp4 and Slp is more efficient using Assembly 2.2 than 2.1. VgrG proteins, red; PAAR proteins or PAAR repeat containing domains, yellow; effector proteins or domains, violet, with the exception of Rhs C-terminal domains which are green or dark blue; conserved T6SS components, blue. Compartments of the secreting cell: OM, outer membrane; PP, periplasm; IM, inner membrane; CP, cytoplasm.

Having considered the roles of the two VgrG homologues, we then proceeded to examine their partners, PAAR proteins. Genetic analysis revealed that three functional VgrG-PAAR combinations exist in *S*. *marcescens*, named Assemblies 1 (VgrG1-Paar1), 2.1 (VgrG2-Rhs1) and 2.2 (VgrG2-Rhs2), schematically depicted in Fig 8. PAAR proteins are proposed to be the equivalent of the phage gp5.4 protein which sits at the tip of gp27-gp5 within the baseplate, and to provide the final sharp ‘tip’ bound to the end of the β-helical domain of VgrG/gp5 proteins [28]. It is essential to have at least one PAAR protein for T6SS activity in our system, and we believe, given the integral nature of the proposed role above, that this finding is likely to be general. In other words, we suggest that at least one PAAR domain-containing protein is essential for a T6SS to function, and thus such proteins can be considered the 14^th^ core component of the T6SS (‘TssN’). The original work identifying PAAR proteins as components of the T6SS showed an important contribution to T6SS function in *V*. *cholerae* but complete essentiality was not seen [28]. However it is possible that further PAAR proteins remain to be discovered in this organism, since it has been reported that cryptic PAAR domains may require detailed analysis to identify [31]. We have shown that PAAR proteins are not required for VgrG stability as proposed [28] but are rather required for secretion of the cognate VgrG, even if another VgrG-PAAR pathway is operational. If no PAAR proteins are present at all, Hcp1 is not secreted, suggesting either that the Hcp tube cannot assemble or the sheath cannot contract when no functional VgrG-PAAR unit is available. This is consistent with the idea that VgrG and PAAR form an integral part of the baseplate, but the precise role and position of this unit, and particularly PAAR proteins, remain to be determined. A very interesting outstanding question is how effector domains fused to PAAR domains (e.g. Rhs repeat-encased toxin domains), and effector proteins bound directly or indirectly to VgrG proteins (e.g. phospholipases), are accommodated within the assembly and spatial arrangement of the baseplate structure and the overall T6SS. Do they ‘hang’ outside the baseplate? Is there a limit to how many can be accommodated in any individual assembly?

In support of the genetic data, we report the isolation of native, stable VgrG2-Rhs-EagR complexes. EagR1 and EagR2 are conserved accessory proteins specifically required for delivery of Rhs1 and Rhs2, respectively (here and [18]). We predicted that EagR proteins should interact with the N-terminal, PAAR-containing domain of their cognate Rhs protein [18] and here were able to show specific Rhs1^NT^-EagR1 and Rhs2^NT^-EagR2 interactions. These data complement a recent study in *P*. *aeruginosa* where a complex containing VgrG1, EagT6, Tse6, Tsi6 and EF-Tu was identified by a similar approach and then characterised by electron microscopy [36]. Tse6 is a specialised effector comprising a PAAR-containing and an NAD(P)^+^ glycohydrolase toxin domain, Tsi6 is its immunity protein, EagT6 is an EagR homologue, and EF-Tu is a cellular protein proposed to mediate entry of Tse6 into the target cell cytoplasm. In this complex, the PAAR repeat domain of Tse6 was mapped to the tip of VgrG1 and EagT6 was localised in proximity to the N-terminal domain of Tse6, which contains the PAAR repeat region and three flanking transmembrane helices. The authors proposed that EagT6 acts as a chaperone protein stabilising this N-terminal domain, since Tse6 is unstable in the absence of EagT6 [36]. Our data are consistent with EagR proteins having a similar chaperone role for Rhs proteins. Each Rhs^NT^ specifically interacts with its own EagR protein and contains two predicted transmembrane helices prior to the PAAR-repeat containing region and the stability of Rhs1 appeared to be reduced in a Δ*eagR1* mutant. We also observed that Rhs2 was no longer associated with VgrG2 in an Δ*eagR2* mutant. However we note that despite the Δ*eagR2* mutant being an in-frame deletion of the gene, negligible *in trans* complementation of its Rhs2-dependent toxicity phenotype could be observed, raising the possibility that loss of the EagR2 coding sequence may, in addition to impaired stabilisation of Rhs2, result in loss of a promoter or translational element ensuring Rhs2 is not expressed in its absence.

In contrast with the Tse6 study [36], we did not detect any co-purification with Rhs of the immunity proteins RhsI1 or RhsI2, or any housekeeping proteins which could have an equivalent role to EF-Tu. This likely reflects a fundamental difference between Rhs-type and other specialised effectors. Our previous data support the idea that the Rhs repeat domain forms a shell-like structure around the C-terminal toxin domain, shielding it from the cytoplasm of the producing cell. Therefore the cytoplasmic immunity protein does not bind the toxin domain in the producing cell, only in resistant recipient cells [18]. Here, the co-purification experiment was performed under conditions where Rhs-containing complexes would only be isolated from producing (pre-secretion) cells and so a shielded toxin domain would explain why neither immunity nor ‘carrier’ cellular proteins were isolated. Other noteworthy proteins that were not detected in the VgrG2 co-purification, even with sensitive mass spectrometry analysis, are VgrG1, Hcp1 and Slp. This implies that VgrG1 and VgrG2 cannot form heterotrimers under native conditions, a conclusion supported by the fact that VgrG1-Paar1 is unable to support secretion of VgrG2 in the absence of Rhs1 and Rhs2. The absence of Hcp1 suggests that Hcp tubes do not stably interact with the VgrG-PAAR tip. This is unexpected, given the current model that an Hcp-VgrG-PAAR structure is propelled from the cell by sheath contraction, but in fact strong evidence for such an interaction is missing for any T6SS to date. We have not been able to detect an interaction between VgrG2 and Slp under physiologically relevant conditions, which may reflect a relatively weak or transient interaction, low levels of the effector or an accessory protein, or even association only during the firing process. On the other hand, whilst EagR1 and EagR2 were readily detected in the VgrG2-associated complex, they were not detected in the secretome. This suggests that they are released inside the secreting cell prior to or during VgrG-PAAR expulsion, in contrast with the model proposed for EagT [36], but similar to the suggestion that Tap-1 accessory proteins may ‘load’ effectors onto VgrG prior to secretion and then be retained inside the cell [34].

Characterisation of the VgrG2 dependent pathways also revealed that efficiency of T6S can depend on the PAAR protein utilised, even with the same VgrG. Specifically, Assembly 2.2 (VgrG2-Rhs2) is more efficient in delivering cargo effectors than Assembly 2.1 (VgrG2-Rhs1) in *S*. *marcescens*. Several reasons for this can be envisaged. It appears that more VgrG2-Rhs2 complex is available in the cell for incorporation into the machinery, since a greater amount of Rhs2 is co-purified with VgrG2 than Rhs1, indicated by ~10^2^-fold greater enrichment values for Rhs2 and EagR2 compared with Rhs1 and EagR1. This could result from the strength of the VgrG2-Rhs2 interaction or the levels of each Rhs protein available. Alternatively, part or all of the T6SS machine could be most stable with the VgrG2-Rhs2 combination. For the VgrG2-specific effector Slp, there could be additional ‘preference’ factors, such as a more optimal spatial interaction with the VgrG2-Rhs2 unit than the VgrG2-Rhs1 unit. The greater efficiency of the VgrG2-Rhs2 unit may be the reason why Rhs2 is widespread, although with highly variable C-terminal domains, across *S*. *marcescens*, whereas Rhs1 is confined to a small subset of strains.

The EagR accessory proteins may also be involved in determining the efficiency of the interactions of different Rhs proteins with their cognate VgrG. Our data indicate that EagR proteins cannot bind to VgrG proteins in the absence of the cognate Rhs. This raises two possibilities: Firstly, the sole role of EagR proteins is as stabilising chaperones for Rhs proteins, perhaps aiding solubility by protecting the hydrophobic helices. This would imply that the only direct interaction of VgrG is with the Rhs PAAR repeat-containing region and the VgrG-Rhs interaction would not depend on EagR if Rhs were stable. Secondly, in addition to EagR’s stabilisation role, the EagR-Rhs^NT^ complex must be recognised by VgrG as a unit, either through interactions with both proteins or because EagR modifies the overall structure of Rhs^NT^ including the PAAR region. In the second scenario, the existence of different EagR proteins may be involved in allowing both Rhs proteins to interact with the same VgrG, given that the N-terminal domains of Rhs1 and Rhs2 are not closely related, belonging to different clades of Rhs protein [18, 48].

In conclusion, this study has allowed us to propose a model for the T6SS of *S*. *marcescens* in which three different VgrG-PAAR assemblies can support a functional secretion system (Fig 8). These assemblies differ in both the effectors that they deploy and the efficiency with which they deliver them. We have highlighted previously unreported nuances of T6SS function, including the use of one VgrG protein by several PAAR proteins and an influence of specific VgrG-PAAR combinations on the efficiency of delivery of Hcp-dependent cargo effectors. Finally, our results allow us to propose that PAAR proteins should be considered the 14^th^ core component of the T6SS and provide further insight into how this versatile secretion machinery is able to deploy multiple and distinct effector toxins using different delivery modes.

## Materials and Methods

### Bacterial strains, plasmids, and culture conditions

Strains and plasmids used in this study are described in S1 Table. Mutant strains of *S*. *marcescens* Db10 carrying in-frame deletions or encoding HA- or His-tagged fusion proteins at the normal chromosomal location were generated by allelic exchange using the pKNG101 suicide vector, and streptomycin-resistant derivatives were generated by phage ϕIF3-mediated transduction, as described previously [12, 41]. Plasmids for constitutive expression of genes *in trans* were derived from pSUPROM and for arabinose-inducible expression from pBAD18-Kn (S1 Table). Details of plasmid construction and primer sequences are given in S2 Table. Strains of *S*. *marcescens* were grown at 30°C in LB (10 g/l tryptone, 5 g/l yeast extract, 10 g/l NaCl, with 1.2 g/l agar for solid media) or Minimal Media (40 mM K_2_HPO_4_, 15 mM KH_2_PO_4_, 0.1% (w/v) (NH_4_)_2_SO_4_, 0.4 mM MgSO_4_, 0.2% (w/v) glucose). Strains of *E*. *coli* were grown in LB at 37°C unless stated otherwise. When required, cultures were supplemented with antibiotics: kanamycin (Kn) 100 μg/ml; streptomycin (Sm) 100 μg/ml, chloramphenicol (Cm) 25 μg/ml or ampicillin (Ap) 100 μg/ml.

### Co-culture assays for T6SS-mediated antibacterial activity

These were based on the assay described previously [12]. Briefly, the attacker strain of *S*. *marcescens* Db10 and appropriate target strain (both at OD_600_ 0.5) were mixed at an initial attacker:target ratio of 1:1 (or 5:1 for *E*. *coli*) and co-cultured on solid LB at 30°C (or 37°C for *E*. *coli*), for either 7.5 h (Db10-derived target strain) or 4 h (other target strains). Surviving target cells were subsequently enumerated by serial dilution and viable counts on streptomycin-supplemented media. The target strain was always the streptomycin-resistant version of the organism or mutant in question (S1 Table). Data are presented as the mean of four independent biological replicates, with standard error of the mean (SEM). When a small difference in activity between two strains was noted (< 10-fold), the significance of the difference was determined by student’s t-test (two-tailed, homoscedastic).

### Immunodetection of intracellular and secreted proteins

Anti-Hcp1, anti-Ssp1 and anti-Ssp2 immunoblots were performed on cultures grown for 7 h as previously described [12, 41]. Whole cell samples for immunodetection of VgrG2-His_6_ and Ssp4-HA were prepared from cultures grown in 25 ml LB for 5 h at 30°C. Cells from 200 μl of culture were resuspended in 100 μl of 2x gel sample buffer (100 mM Tris-HCl pH 6.8, 3.2% SDS, 3.2 mM EDTA, 16% glycerol, 0.2 mg/ml bromophenol blue, 2.5% β-mercaptoethanol) and boiled for 10 min. Secreted proteins were precipitated from 25 ml of culture supernatant using chloroform:methanol as described [12] and resuspended in 100 μl 2x sample buffer. Anti-His (Qiagen), anti-HA (Generon) and anti-RNAP-β (Neoclone) primary antibodies were used at 1:6,000, 1:10,000 and 1:20,000, respectively, with peroxidase-conjugated anti-mouse secondary antibody (Roche) at 1:10,000.

### Proteomic secretome sample preparation

Four independent cultures of *S*. *marcescens* Db10, Δ*vgrG1*, Δ*vgrG2* and Δ*vgrG1*Δ*vgrG2* mutants were grown in 200 ml Minimal Media with vigorous agitation for 8 h to an OD_600_ of 1.2. Culture supernatant was collected and separated from the cells by five rounds of centrifugation at 5000 *g* for 30 min at 4°C. Total protein from 40 ml of culture supernatant was precipitated overnight on ice with 6.25% (w/v) trichloroacetic acid (Sigma), recovered by centrifugation (5000 *g*, 4°C, 30 min), washed five times in 1 ml ice-cold 80% (v/v) acetone and air dried. Precipitated proteins were redissolved in 1% sodium 3-[(2-methyl-2-undecyl-1,3-dioxolan-4-yl)methoxy]-1-propanesulfonate (commercially available as RapiGest, Waters), 50 mM Tris-HCl pH 8.0, 1 mM TCEP, prior to mass spectrometry.

### Affinity isolation of chromosomally His_6_-tagged VgrG2 protein

*S*. *marcescens* Db10 and FRC17 (VgrG2-His_6_) were grown in 50 ml of LB with vigorous agitation for 4 h. Cells were harvested by centrifugation at 4000 *g* for 20 min and washed with 50 mM Tris-HCl, pH 7.5. Cell pellets were resuspended in 800 μl of lysis buffer (BPER [Thermo Scientific] supplemented with Complete Mini EDTA-free Protease Inhibitor Cocktail [Roche], DNaseI, 0.1 mg/ml lysozyme, 20 mM imidazole) and incubated for 15 min at 40 rpm (end-over-end rotation). Lysates were centrifuged at 14000 *g* for 20 min at 4°C to remove cell debris, added to 30 μl of magnetic Ni-NTA (Ni^2+^-nitrilotriacetate) beads (Qiagen) previously washed with 5x 1 ml wash buffer (20 mM Tris-HCl, pH 7.5, 100 mM NaCl, 50 mM imidazole, 0.1% Triton X-100), and incubated for 1 h at 4°C at 40 rpm. The beads were then washed with 5x 1 ml wash buffer and bound proteins were eluted by the addition of 20 μl 2x gel sample buffer (with 2.5% β-mercaptoethanol) and boiled for 2 min. Eluted proteins were resolved and analysed by 15% SDS-PAGE and colloidal Coomassie Blue staining. For initial identification of interacting partners, bands of interest were excised from the SDS-PAGE gel and the proteins identified by 1D nanoLC-MS-MS at the FingerPrints Proteomics Facility, University of Dundee. For quantitative identification of VgrG2-His_6_-interacting proteins, affinity purifications were performed as above on three independent biological replicates of each strain. Proteins were eluted from the Ni-NTA beads in 40 μl 3% (w/v) RapiGest, 50 mM Tris-HCl pH 8.0, 1 mM TCEP prior to mass spectrometry.

### Mass spectrometry and label-free quantitation

For protein samples prepared as above, cysteines were alkylated by addition of 20 mM iodoacetamide and incubation for 20 min at 25°C in the dark and then the reaction was quenched by addition of 20 mM DTT (5 mM iodoacetamide and 5 mM DTT were used for the VgrG2-His_6_ co-purification samples). Samples were diluted to 0.1% Rapigest with 50 mM Tris-HCl pH 8.0, and Trypsin (sequencing grade, Promega) was added at a 1:50 ratio. Proteins were digested overnight at 37°C under constant shaking.

Samples, either 0.5 μg of digest from four biological replicates for the secretome analysis or 20% of eluted peptides from three biological replicates for the VgrG2-His_6_ co-purification analysis, were injected in an interleaved manner onto a 2 cm x 100 μm trap column and separated on a 15 cm x 75 μm Pepmap C18 reversed-phase column (both Dionex, now Thermo Fisher Scientific) on a Dionex 3000 Ultimate RSLC. Peptides were eluted by a linear 2 h gradient (1 h gradient for co-purification samples) of 95% A/5% B to 35% B (A: H_2_O, 0.1% Formic acid (FA); B: 80% ACN, 0.08% FA) at 300 nl/min into a LTQ Orbitrap Velos (Thermo-Fisher Scientific). Data was acquired using a data-dependent “top 20” method, dynamically choosing the most abundant precursor ions from the survey scan (400–1600 Th, 60,000 resolution, AGC target value 10^6^). Precursors above the threshold of 2000 counts were isolated within a 2 Th window and fragmented by CID in the LTQ Velos using normalised collision energy of 35 and an activation time of 10 ms. Dynamic exclusion was defined by a list size of 500 features and exclusion duration of 60 s. Lock mass was used and set to 445.120025 for ions of polydimethylcyclosiloxane (PCM).

Label-free quantitation was performed using MaxQuant 1.5.1.7 [49]. Mass spectrometric runs of four biological replicates of Db10 (WT), Δ*vgrG*, Δ*vgrG2* and Δ*vgrG1*Δ*vgrG2* were searched against a combined database of *Serratia marcescens* Db10 containing 4,720 sequences and a list of common contaminants in proteomics experiments using the following settings: enzyme Trypsin/P, allowing for 2 missed cleavage, fixed modifications were carbamidomethyl (C), variable modifications were set to Acetyl (Protein N-term), Deamidation (NQ) and Oxidation (M). MS/MS tolerance was set to 0.5 Da, precursor tolerance was set to 6 ppm. Peptide and Protein FDR was set to 0.01, minimal peptide length was 7, and one unique peptide was required. Re-quantify and retention time alignment (2 min) were enabled. A student’s t-test (two-tailed, homoscedastic) or a two-way ANOVA was performed on the LFQ intensities and proteins with p<0.05 and a fold-change >4-fold were considered significantly altered in abundance.

### Bacterial two-hybrid analysis

Bacterial two-hybrid analyses were performed following established protocols [43, 50]. *E*. *coli* MG1655 Δ*cyaA* was co-transformed with combinations of a pUT18-based and a pT25-based plasmid and the color of the resulting transformants scored on MacConkey media with Ap, Cm and 0.2% maltose (positive result being red). For quantitative measurement of the interaction, β-galactosidase assays were performed as described [12] on double-transformed MG1655 Δ*cyaA* grown at 30°C in LB and permeabilized with toluene. Replicate assays were performed on three independent transformants.

## Supporting Information

S1 Fig The Type VI system of *S*. *marcescens* Db10 has two distinct VgrG homologues. (A) Pairwise sequence alignment of VgrG1 (SMDB11_2244) and VgrG2 (SMDB11_2276) performed using the EMBOSS Needle algorithm (www.ebi.ac.uk). (B) Number of recovered cells of target organisms *E*. *coli* MC4100 and *S*. *marcescens* ATCC274 following co-culture with wild type (WT) or mutant (Δ*tssE*, Δ*vgrG1*, Δ*vgrG2* and Δ*vgrG1*Δ*vgrG2*) strains of *S*. *marcescens* Db10 as attacker. Points show mean +/- SEM (n = 3 or 4). The grey point for the Δ*vgrG1* mutant indicates that recovery was below the detection limit of the assay, in other words ≤ 10 cells per co-culture spot.
(TIF)

S2 Fig Hcp1 is essential for Type VI secretion function and Ssp4 delivery in *S*. *marcescens* Db10. (A) Immunoblot detection of Hcp1 and Ssp2 in cellular and secreted fractions of wild type (WT), mutant strains Δ*tssE*, Δ*hcp1* (Δ*SMDB11_2263*), Δ*hcp2* (Δ*SMDB11_3455*) or Δ*hcp3* (Δ*SMDB11_3456*), and wild type or mutant strains carrying either the vector control plasmid (+VC, pSUPROM) or a plasmid directing the expression of Hcp1 (+Hcp1, pSC715) *in trans*. (B)-(D) Recovery of target strains *P*. *fluorescens* or *S*. marcescens Db10 *Δssp4Δsip4* (susceptible to Ssp4), following co-culture with wild type, mutant, or complemented strains as attacker. Points show mean ± SEM (n = 4).
(TIF)

S3 Fig Multiscatter plot of label-free secretomics data. Label-free intensities (in log2) of four biological replicates of *S*. *marcescens* Db10 (WT), Δ*vgrG2*, Δ*vgrG1* and Δ*vgrG1*Δ*vgrG2* (Δ*G1G2*) are plotted against each other, demonstrating high levels of reproducibility between samples.
(TIF)

S4 Fig Type VI secretion system activity and complementation of Δ*slp* and related strains of *S*. *marcescens* Db10. (A) Immunoblot detection of Hcp1 and Ssp2 in cellular and secreted fractions of the wild type (WT), mutants Δ*tssE*, Δ*slp* (Δ*SMDB11_0927*) and Δ*0927–0929* (Δ*SMDB11_0927*–*0929*), and the strain encoding a VgrG2-His_6_ fusion protein at the normal chromosomal location in a Δ*vgrG1* background (Δ*vgrG1*, VgrG2-His). (B) Recovery of target strain susceptible to Slp (Δ*0927–0929*, Δ*SMDB11_0927*–*0929* carrying pSUPROM) following co-culture with wild type or mutant strains (Δ*tssE* or Δ*slp)* carrying either the vector control plasmid (+ VC, pSUPROM) or a plasmid directing the expression of Slp (+ Slp, pSC772).
(TIF)

S5 Fig Delivery of VgrG2-specific effectors Rhs1, Rhs2 and Slp is not impaired in mutants lacking Paar1. Recovery of target strains *S*. *marcescens* Db10 Δ*tssH*Δ*rhsI1*, Δ*rhs2*Δ*rhsI2* or Δ*0927–0929* (susceptible to Rhs1-, Rhs2- or Slp-dependent anti-bacterial activity, respectively) following co-culture with wild type (WT) or mutant (Δ*tssE*, Δ*paar1* and Δ*paar1*Δ*vgrG1*) strains as attacker. Points show mean ± SEM (n = 4). The data shown for the Δ*tssH*Δ*rhsI1* and Δ*rhs2*Δ*rhsI2* targets is part of the same experiments as shown in Fig 2 and the WT and Δ*tssE* data points are repeated from that figure.
(TIF)

S6 Fig Specificity and self-interaction of EagR proteins. (A) Bacterial two-hybrid analysis of all combinations of interactions between EagR1 and the N-terminal domain of Rhs1 (left) and all combinations of interactions between EagR1 and EagR2 (right). Each protein was fused with either the T18 or T25 domain of CyaA as indicated. Negative controls were provided by the empty vectors, pUT18 and pT25 (-), and a positive control by the self-interaction of TssK (+ve). Shown is the β-galactosidase activity of the reporter strain transformed with the combinations of plasmids indicated. Bars show mean +/- SEM (n = 3 independent transformations). The data for the left graph are part of the same experiment as Fig 7C and some of the data points are repeated between the two figures; the right graph shows an independent experiment. (B) EagR2 is a specific accessory protein required for Rhs2-dependent anti-bacterial activity. Recovery of target strains lacking either *rhsI2* (*S*. *marcescens* Db10 Δ*rhs2*Δ*rhsI2*), left, or *rhsI1* (*S*. *marcescens* Db10 Δ*rhsI1*Δ*tssH*), right, following co-culture with wild type or mutant (Δ*tssE*, Δ*rhs1*, Δ*eagR1*, Δ*rhs2* and Δ*eagR2*) strains of Db10 as attacker. Points show mean ± SEM (n = 4).
(TIF)

S7 Fig Rhs stability and association with VgrG2 is compromised in *eagR* mutants. (A) Affinity isolation of VgrG2-His and co-purifying proteins from strains of *S*. *marcescens*. As Fig 7A, except that the VgrG2-His affinity purification was performed in a strain lacking EagR2 (Δ*rhs1*Δ*eagR2*), together with appropriate control strains (parental, Δ*rhs1* and Δ*rhs1*Δ*rhs2*). (B) Cellular levels of Rhs1 fused at its N-terminus with a triple FLAG epitope tag (FLAG-Rhs1) detected by anti-FLAG immunoblot. Strains analysed were wild type *S*. *marcescens* Db10 with FLAG-Rhs1 encoded at the normal chromosomal location (chrom. FLAG-Rhs1) and the wild type control with no fusion (WT), and the wild type or Δ*eagR1* mutant carrying either vector control (+VC, pBAD18-Kn) or plasmid directing the inducible expression of FLAG-Rhs1 *in trans* (+pFLAG-Rhs1, pSC697). Levels of l-arabinose (inducer) are given for the strains expressing plasmid-borne FLAG-Rhs1 and RNAP was also detected as control cellular protein. Samples were normalised such that cellular protein from the same number of cells was loaded for chromosomal and plasmid fusions and two different exposures of the anti-FLAG blot are presented. Note that FLAG-Rhs1 has a predicted MW of 167 kDa but the fusion protein is detected with an apparent MW of around 35 kDa from both chromosomal and plasmid locations. It is unclear whether this is due to altered mobility or cleavage, however either way, since the epitope tag is N-terminal, the protein detected should include the N-terminal PAAR repeat containing region predicted to be stabilised by EagR1.
(TIF)

S1 Dataset Full label-free secretomics data. Sheet 1, All data. Sheet 2, Proteins significantly upregulated in the secretome of the Δ*vgrG1*Δ*vgrG2* mutant compared with the wild type.
(XLSX)

S1 Table Bacterial strains and plasmids. (PDF)

S2 Table Oligonucleotide primers for plasmid construction. (PDF)
