# Supplementary figures and images for: VgrG and PAAR Proteins Define Distinct Versions of a Functional Type VI Secretion System
Source: PLoS Pathog. 2016 Jun 28;12(6):e1005735. doi: 10.1371/journal.ppat.1005735 (PMC4924876; doi:10.1371/journal.ppat.1005735)

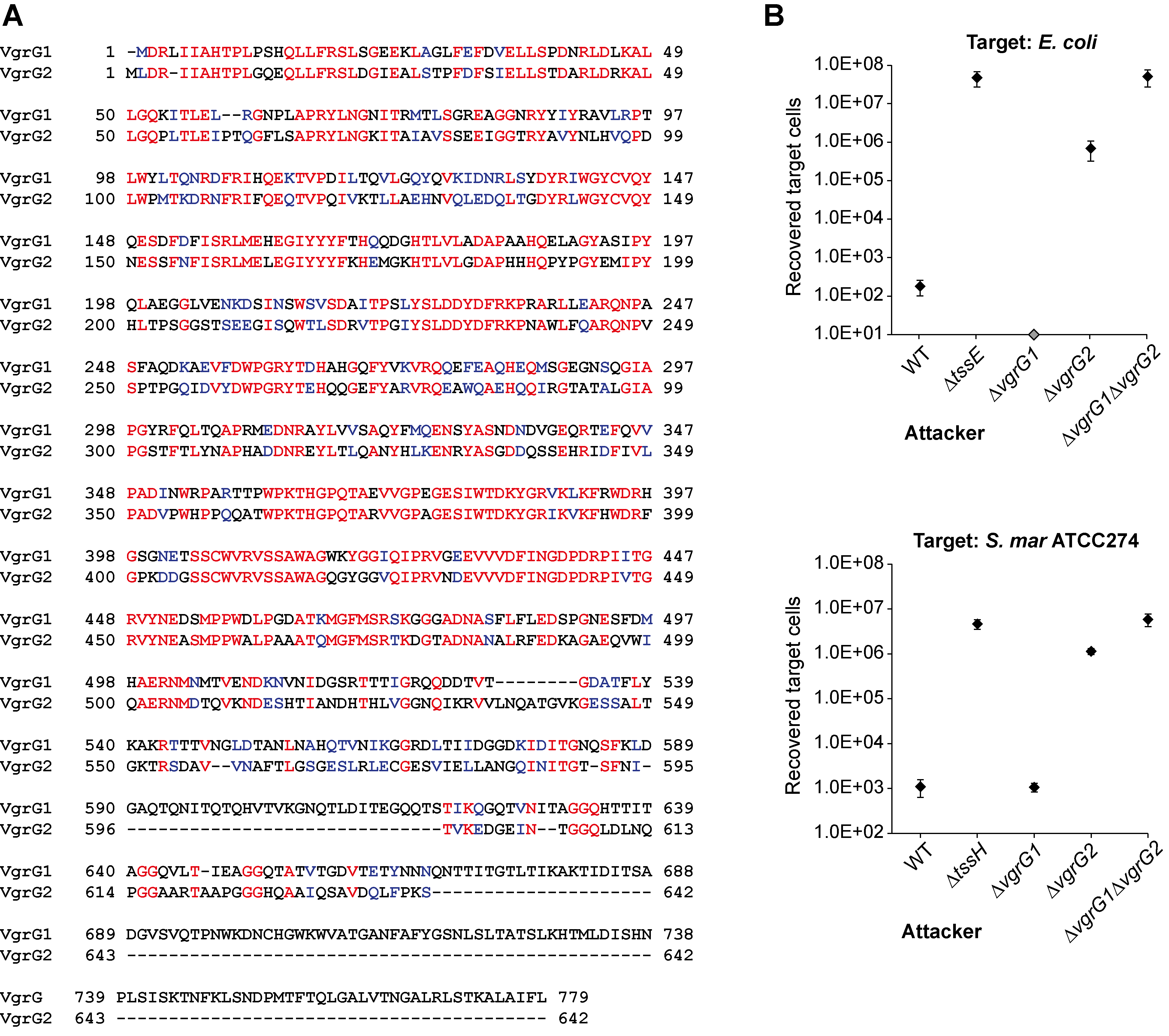

Supplement: S1 Fig — (A) Pairwise sequence alignment of VgrG1 (SMDB11_2244) and VgrG2 (SMDB11_2276) performed using the EMBOSS Needle algorithm (www.ebi.ac.uk). (B) Number of recovered cells of target organisms E. coli MC4100 and S. marcescens ATCC274 following co-culture with wild type (WT) or mutant (ΔtssE, ΔvgrG1, ΔvgrG2 and ΔvgrG1ΔvgrG2) strains of S. marcescens Db10 as attacker. Points show mean +/- SEM (n = 3 or 4). The grey point for the ΔvgrG1 mutant indicates that recovery was below the detection limit of the assay, in other words ≤ 10 cells per co-culture spot. (TIF) [file ppat.1005735.s001.tif]

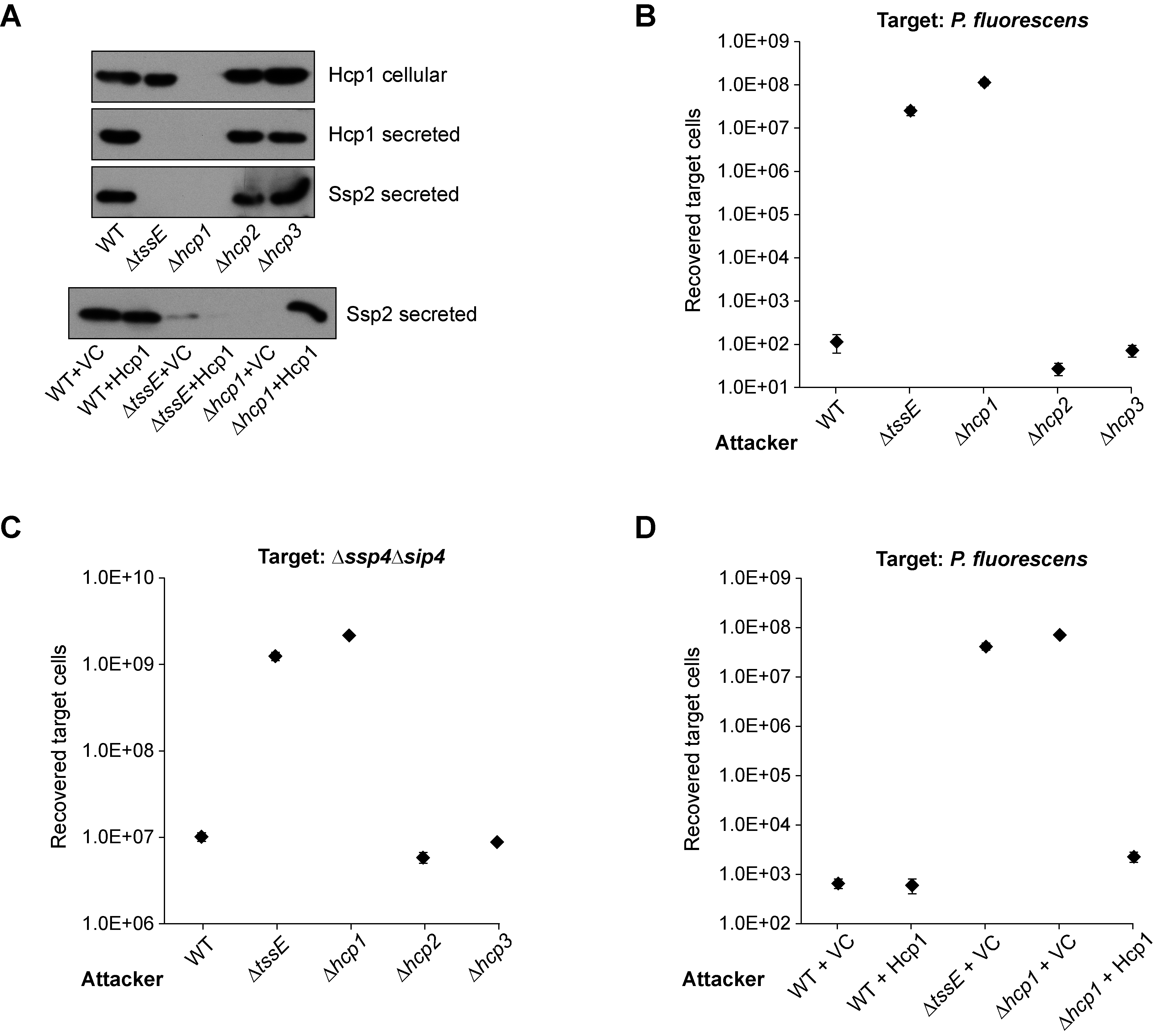

Supplement: S2 Fig — (A) Immunoblot detection of Hcp1 and Ssp2 in cellular and secreted fractions of wild type (WT), mutant strains ΔtssE, Δhcp1 (ΔSMDB11_2263), Δhcp2 (ΔSMDB11_3455) or Δhcp3 (ΔSMDB11_3456), and wild type or mutant strains carrying either the vector control plasmid (+VC, pSUPROM) or a plasmid directing the expression of Hcp1 (+Hcp1, pSC715) in trans. (B)-(D) Recovery of target strains P. fluorescens or S. marcescens Db10 Δssp4Δsip4 (susceptible to Ssp4), following co-culture with wild type, mutant, or complemented strains as attacker. Points show mean ± SEM (n = 4). (TIF) [file ppat.1005735.s002.tif]

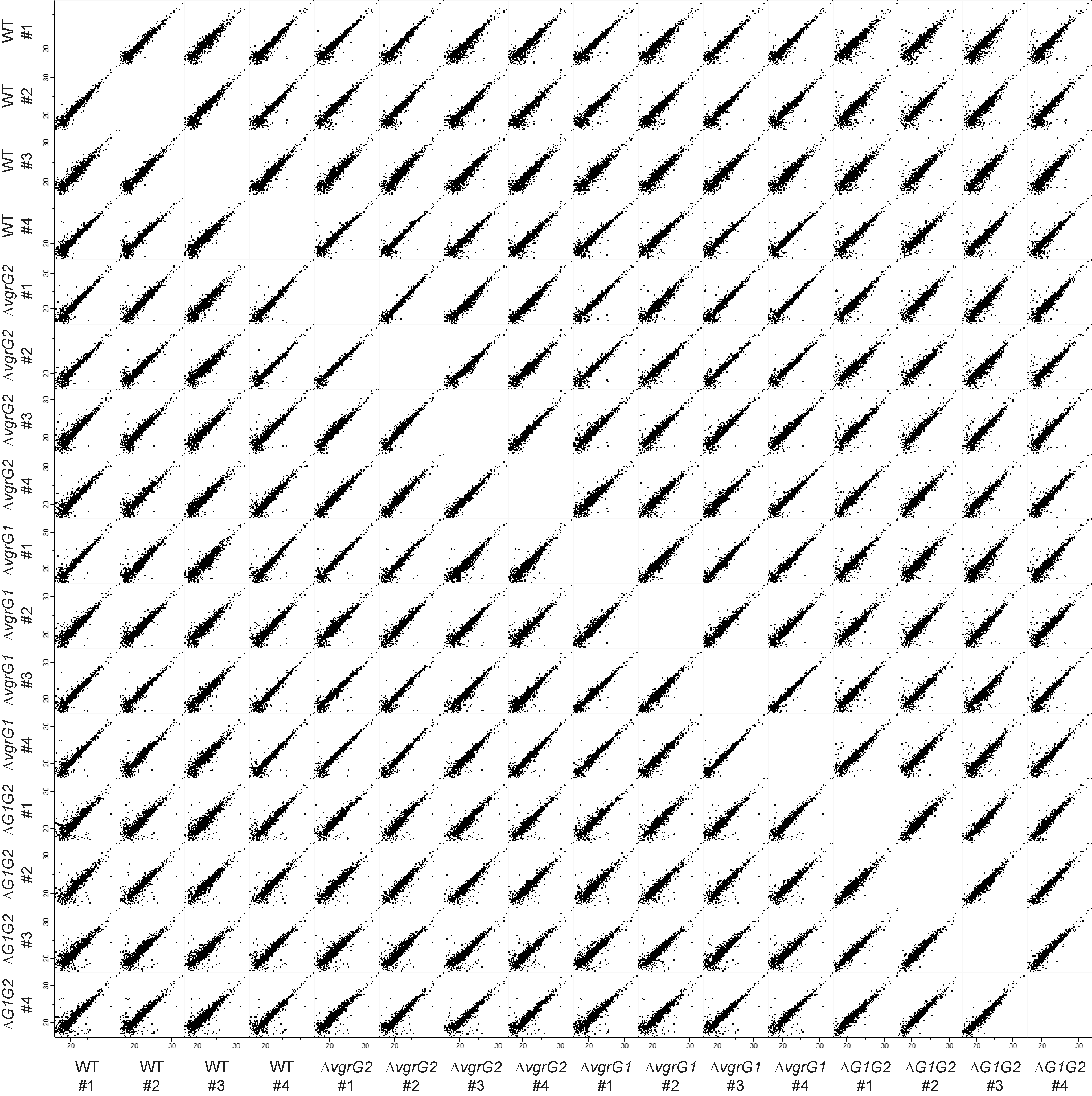

Supplement: S3 Fig — Label-free intensities (in log2) of four biological replicates of S. marcescens Db10 (WT), ΔvgrG2, ΔvgrG1 and ΔvgrG1ΔvgrG2 (ΔG1G2) are plotted against each other, demonstrating high levels of reproducibility between samples. (TIF) [file ppat.1005735.s003.tif]

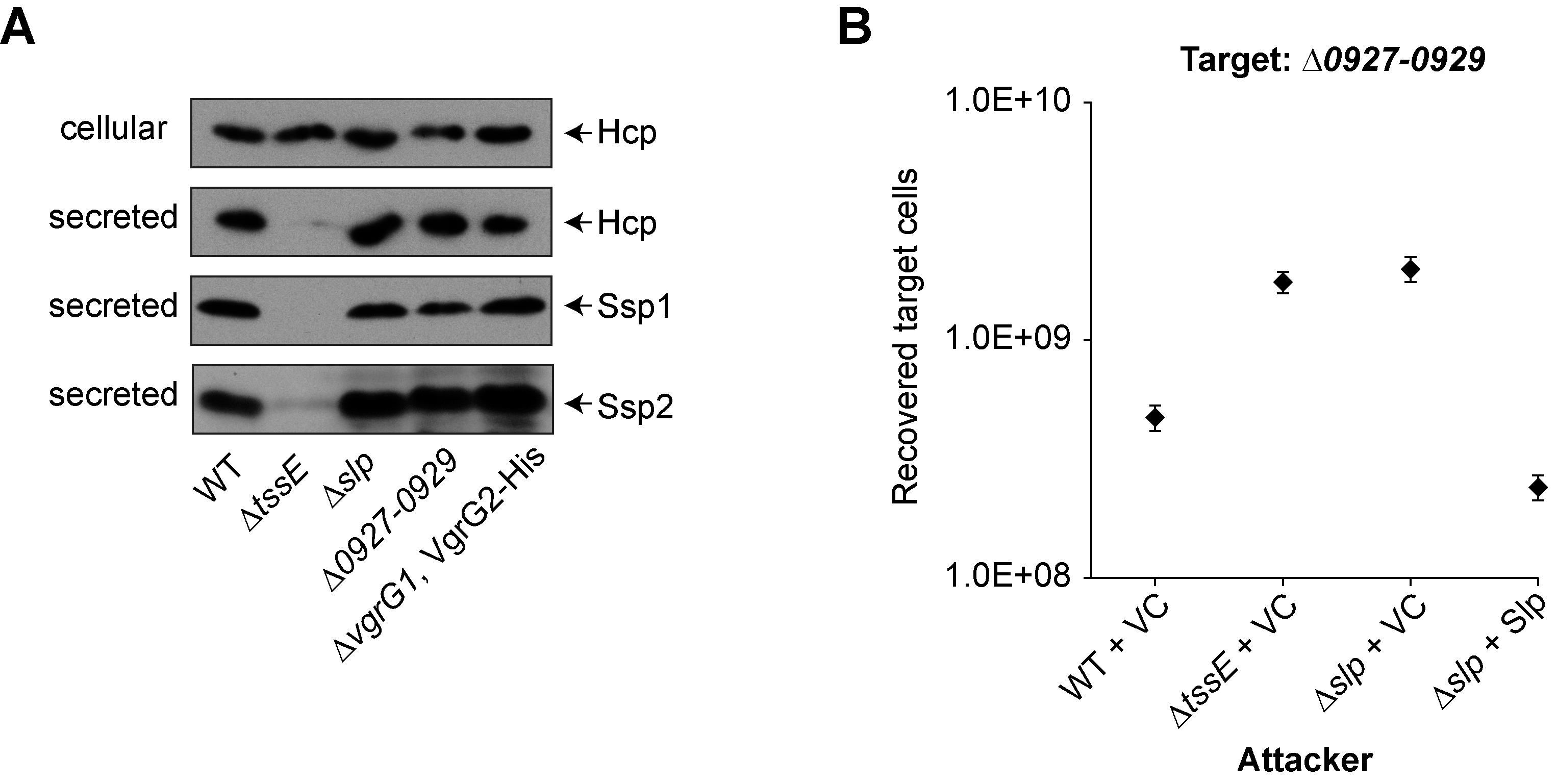

Supplement: S4 Fig — (A) Immunoblot detection of Hcp1 and Ssp2 in cellular and secreted fractions of the wild type (WT), mutants ΔtssE, Δslp (ΔSMDB11_0927) and Δ0927–0929 (ΔSMDB11_0927–0929), and the strain encoding a VgrG2-His6 fusion protein at the normal chromosomal location in a ΔvgrG1 background (ΔvgrG1, VgrG2-His). (B) Recovery of target strain susceptible to Slp (Δ0927–0929, ΔSMDB11_0927–0929 carrying pSUPROM) following co-culture with wild type or mutant strains (ΔtssE or Δslp) carrying either the vector control plasmid (+ VC, pSUPROM) or a plasmid directing the expression of Slp (+ Slp, pSC772). (TIF) [file ppat.1005735.s004.tif]

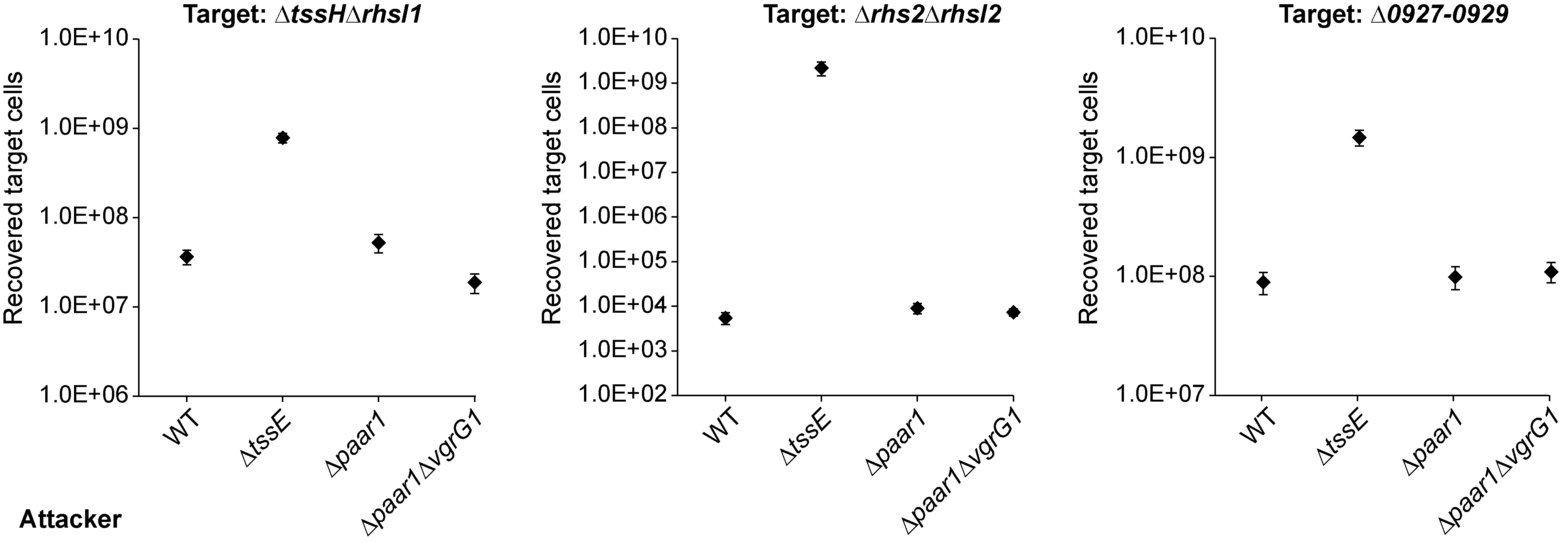

Supplement: S5 Fig — Recovery of target strains S. marcescens Db10 ΔtssHΔrhsI1, Δrhs2ΔrhsI2 or Δ0927–0929 (susceptible to Rhs1-, Rhs2- or Slp-dependent anti-bacterial activity, respectively) following co-culture with wild type (WT) or mutant (ΔtssE, Δpaar1 and Δpaar1ΔvgrG1) strains as attacker. Points show mean ± SEM (n = 4). The data shown for the ΔtssHΔrhsI1 and Δrhs2ΔrhsI2 targets is part of the same experiments as shown in Fig 2 and the WT and ΔtssE data points are repeated from that figure. (TIF) [file ppat.1005735.s005.tif]

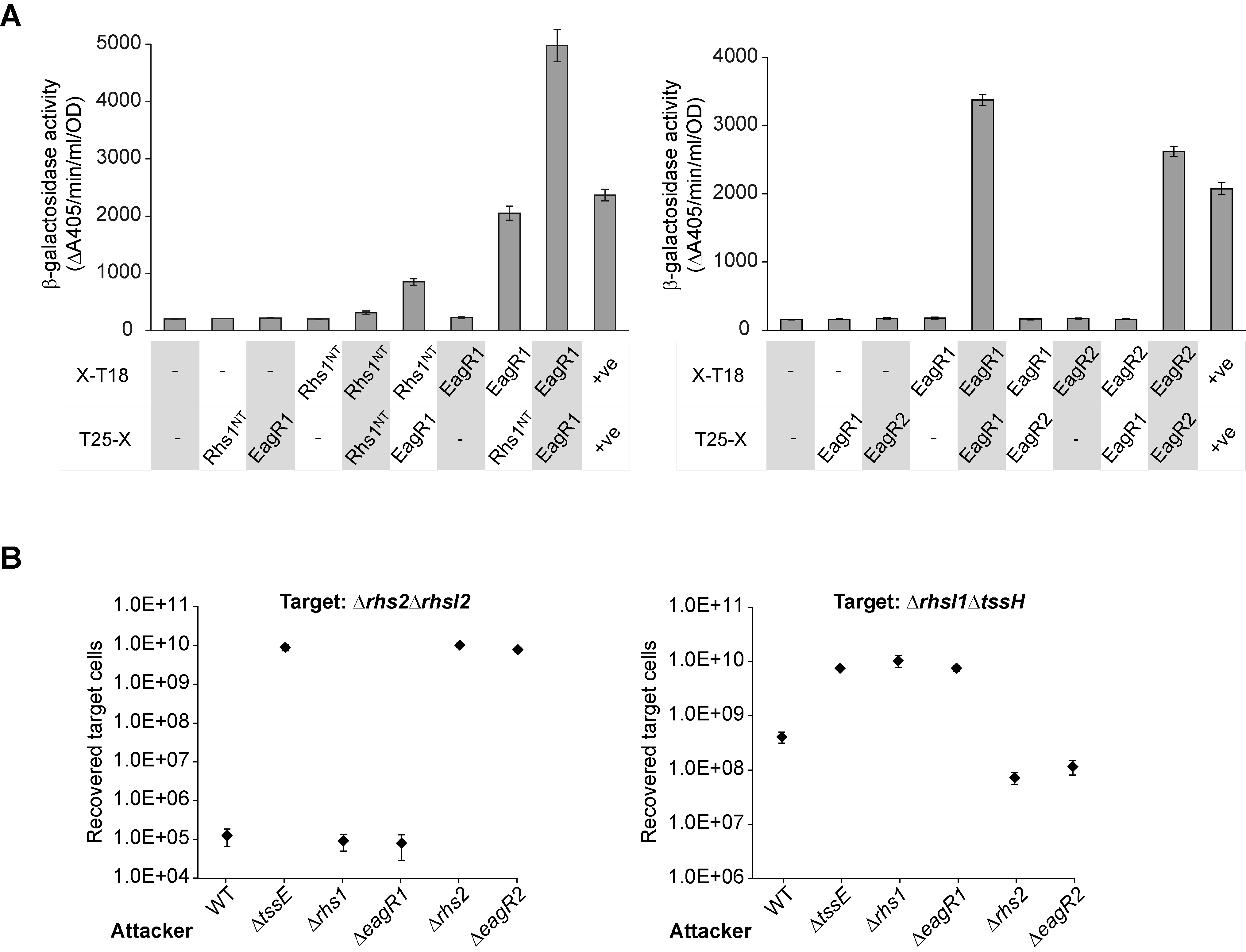

Supplement: S6 Fig — (A) Bacterial two-hybrid analysis of all combinations of interactions between EagR1 and the N-terminal domain of Rhs1 (left) and all combinations of interactions between EagR1 and EagR2 (right). Each protein was fused with either the T18 or T25 domain of CyaA as indicated. Negative controls were provided by the empty vectors, pUT18 and pT25 (-), and a positive control by the self-interaction of TssK (+ve). Shown is the β-galactosidase activity of the reporter strain transformed with the combinations of plasmids indicated. Bars show mean +/- SEM (n = 3 independent transformations). The data for the left graph are part of the same experiment as Fig 7C and some of the data points are repeated between the two figures; the right graph shows an independent experiment. (B) EagR2 is a specific accessory protein required for Rhs2-dependent anti-bacterial activity. Recovery of target strains lacking either rhsI2 (S. marcescens Db10 Δrhs2ΔrhsI2), left, or rhsI1 (S. marcescens Db10 ΔrhsI1ΔtssH), right, following co-culture with wild type or mutant (ΔtssE, Δrhs1, ΔeagR1, Δrhs2 and ΔeagR2) strains of Db10 as attacker. Points show mean ± SEM (n = 4). (TIF) [file ppat.1005735.s006.tif]

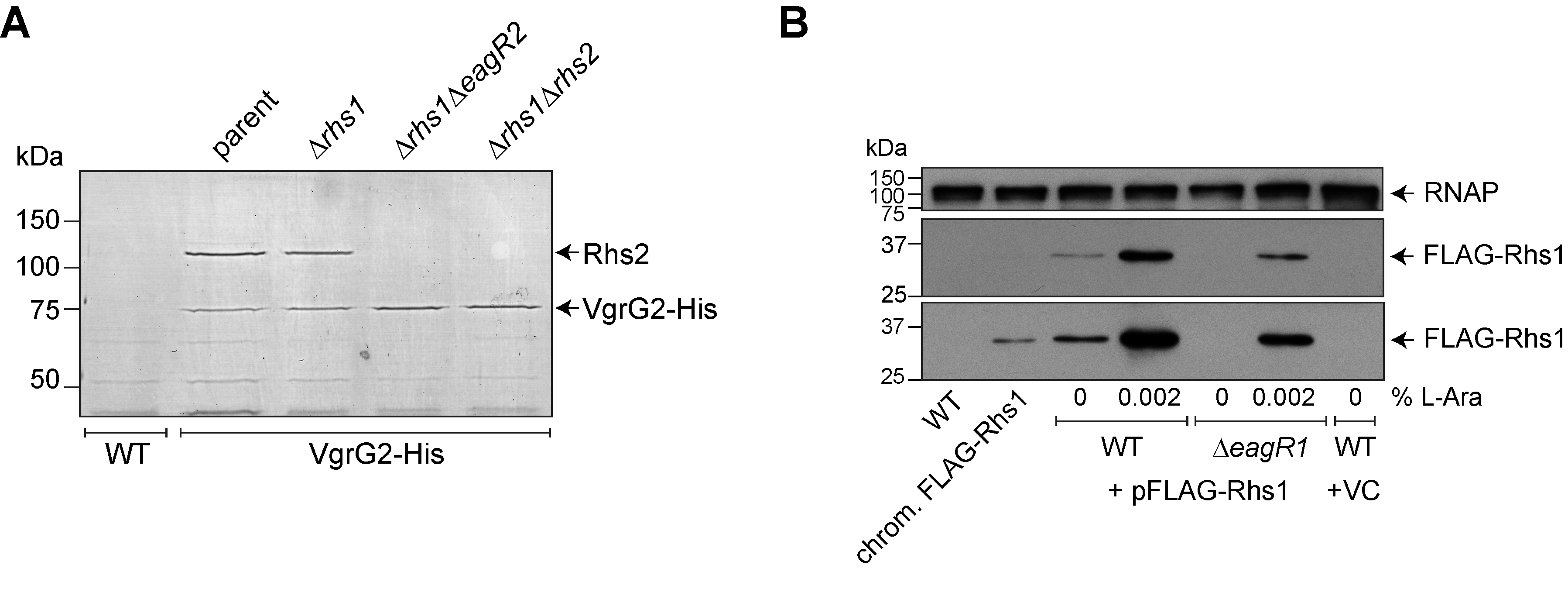

Supplement: S7 Fig — (A) Affinity isolation of VgrG2-His and co-purifying proteins from strains of S. marcescens. As Fig 7A, except that the VgrG2-His affinity purification was performed in a strain lacking EagR2 (Δrhs1ΔeagR2), together with appropriate control strains (parental, Δrhs1 and Δrhs1Δrhs2). (B) Cellular levels of Rhs1 fused at its N-terminus with a triple FLAG epitope tag (FLAG-Rhs1) detected by anti-FLAG immunoblot. Strains analysed were wild type S. marcescens Db10 with FLAG-Rhs1 encoded at the normal chromosomal location (chrom. FLAG-Rhs1) and the wild type control with no fusion (WT), and the wild type or ΔeagR1 mutant carrying either vector control (+VC, pBAD18-Kn) or plasmid directing the inducible expression of FLAG-Rhs1 in trans (+pFLAG-Rhs1, pSC697). Levels of l-arabinose (inducer) are given for the strains expressing plasmid-borne FLAG-Rhs1 and RNAP was also detected as control cellular protein. Samples were normalised such that cellular protein from the same number of cells was loaded for chromosomal and plasmid fusions and two different exposures of the anti-FLAG blot are presented. Note that FLAG-Rhs1 has a predicted MW of 167 kDa but the fusion protein is detected with an apparent MW of around 35 kDa from both chromosomal and plasmid locations. It is unclear whether this is due to altered mobility or cleavage, however either way, since the epitope tag is N-terminal, the protein detected should include the N-terminal PAAR repeat containing region predicted to be stabilised by EagR1. (TIF) [file ppat.1005735.s007.tif]
