# Supplementary material for: VgrG and PAAR Proteins Define Distinct Versions of a Functional Type VI Secretion System
Source: PLoS Pathog. 2016 Jun 28;12(6):e1005735. doi: 10.1371/journal.ppat.1005735 (PMC4924876; doi:10.1371/journal.ppat.1005735)
Supplement: S1 Table — (PDF) [file ppat.1005735.s009.pdf]

S1 Table. Bacterial strains and plasmids used in this study.

Name	Description	Source / Reference
Strains		
Serratia marcescens		
Db10	Wild type	[1]
SJC3	Db10 Δ*tssH* (Δ*SMDB11_2274*)	[2]
SJC11	Db10 Δ*tssE* (Δ*SMDB11_2271*)	[2]
FRA02	Db10 Δ*vgrG1* (Δ*SMDB11_2244*)	This study
FRA01	Db10 Δ*vgrG2* (Δ*SMDB11_2276*)	This study
FRA03	Db10 Δ*vgrG1*Δ*vgrG2*	This study
FRC32	Db10 Δ*paar1* (Δ*SMDB11_2250*)	This study
MJF15	Db10 Δ*rhs1* (Δ*SMDB11_2278*)	[3]
JAD13	Db10 Δ*rhs2* (Δ*SMDB11_1610*)	[3]
JAD14	Db10 Δ*rhs1*Δ*rhs2*	[3]
FRC25	Db10 Δ*paar*Δ*rhs1*Δ*rhs2*	This study
FRC27	Db10 Δ*vgrG1*Δ*paar1*	This study
FRC26	Db10 Δ*vgrG2*Δ*paar1*	This study
FRC28	Db10 Δ*vgrG1*Δ*rhs1*	This study
FRC29	Db10 Δ*vgrG1*Δ*rhs2*	This study
FRC30	Db10 Δ*vgrG1*Δ*rhs1*Δ*rhs2*	This study
FRC31	Db10 Δ*vgrG2*Δ*rhs1*Δ*rhs2*	This study
JAD12	Db10 Δ*eagR1* (Δ*SMDB11_2277*)	[3]
JAD34	Db10 Δ*eagR2* (Δ*SMDB11_1609*)	This study
FRC06	Db10 Δ*hcp1* (Δ*SMDB11_2263*)	This study
FRC07	Db10 Δ*hcp2* (Δ*SMDB11_3455*)	This study
FRC08	Db10 Δ*hcp3* (Δ*SMDB11_3456*)	This study
FRC21	Db10 VgrG1-His (encodes VgrG1 [SMDB11_2244] with a C-terminal His_6 tag at the native chromosomal location)	This study
FRC17	Db10 VgrG2-His (encodes VgrG2 [SMDB11_2276] with a C-terminal His_6 tag at the native chromosomal location)	This study
FRC37	Db10 VgrG1-His, Δ*vgrG2* (encodes VgrG1 with a C-terminal His_6 tag in a Δ*vgrG2* background)	This study
FRC38	Db10 VgrG2-His, Δ*vgrG1* (encodes VgrG2 with a C-terminal His_6 tag in a Δ*vgrG1* background)	This study
FRC55	Db10 VgrG2-His, Δ*tssE* (encodes VgrG2 with a C-terminal His_6 tag in a Δ*tssE* background)	This study
FRC56	Db10 VgrG2-His, Δ*rhs1* (encodes VgrG2 with a C-terminal His_6 tag in a Δ*rhs1* background)	This study
FRC57	Db10 VgrG2-His, Δ*rhs2* (encodes VgrG2 with a C-terminal His_6 tag in a Δ*rhs2* background)	This study
FRC58	Db10 VgrG2-His, Δ*rhs1*Δ*rhs2* (encodes VgrG2 with a C-terminal His_6 tag in a Δ*rhs1*Δ*rhs2* background)	This study
JAD35	Db10 VgrG2-His, Δ*rhs1*Δ*eagR2* (encodes VgrG2 with a C-terminal His_6 tag in a Δ*rhs1*Δ*eagR2* background)	This study
FRC41	Db10 Ssp4-HA (encodes Ssp4 [SMDB11_3980] with a C-terminal HA tag at the native chromosomal location)	This study

FRC53	Db10 Ssp4-HA, Δ*tssE* (encodes Ssp4 with a C-terminal HA tag in a Δ*tssE* background)	This study
FRC54	Db10 Ssp4-HA, Δ*tssE*Δ*hcp1* (encodes Ssp4 with a C-terminal HA tag in a Δ*tssE*, Δ*hcp1* [Δ*SMDB11_2263*] background)	This study
JAD18	Db10 FLAG-Rhs1 (encodes Rhs1 with an N-terminal 3xFLAG tag at the native chromosomal location)	This study
FRC34	Db10 Δ*slp* (Δ*SMDB11_0927*)	This study
FRC39	Db10 Δ*0927-0929* (Δ*SMDB11_0927-SMDB11_0929*)	This study
FRC40	Sm-resistant derivative of FRC39 (Δ*0927-0929*)	This study
JAD09	Db10 Δ*rhsI1*Δ*tssH*, Sm-resistant (Rhs1-susceptible target strain)	[3]
JAD17	Db10 Δ*rhs2*Δ*rhsI2*, Sm-resistant (Rhs2-susceptible target strain)	[3]
KT63	Db10 Δ*ssp2*Δ*rap2a*, Sm-resistant (Ssp2-susceptible target strain)	[4]
JAD06	Db10 Δ*ssp4*Δ*sip4*, Sm-resistant (Ssp4-susceptible target strain)	[5]
SJC17	Sm-resistant derivative of *S. marcescens* ATCC 274	[2]
Pseudomonas fluorescens		
KT02	Sm-resistant derivative of *P. fluorescens* 55	[2]
Escherichia coli		
MC4100	Model K-12 strain; Sm-resistant (*rpsL150*)	[6]
CC118λ*pir*	Cloning host and donor strain for pKNG101-derived allelic exchange plasmids (λ*pir*)	[7]
HH26 pNJ5000	Mobilizing strain for conjugal transfer	[8]
	MG1655 Δ*cyaA*::*apr*	F. Sargent

Plasmids

pKNG101	Suicide vector for allelic exchange (Sm^{R} *sacBR mobRK2 oriR6K*)	[9]
pSUPROM	Vector for constitutive expression of cloned genes under the control of the *E. coli tat* promoter (Kn^{R})	[10]
pBAD18-Kn	Vector for arabinose-inducible expression of cloned genes under the control of the P$_{ara}$ promoter.	[11]
pUT18	Bacterial Two Hybrid plasmid (for fusion of protein of interest with C-terminal T18 fragment of CyaA; Ap^{R})	[12]
pT25	Bacterial Two Hybrid plasmid (for fusion of protein of interest with N-terminal T25 fragment of CyaA; Cm^{R})	[13]
pSC601	pKNG101-derived allelic exchange plasmid for the generation of chromosomal in-frame Δ*SMDB11_2276* (Δ*vgrG2*) deletion	This study
pSC602	pKNG101-derived allelic exchange plasmid for the generation of chromosomal in-frame Δ*SMDB11_2244* (Δ*vgrG1*) deletion	This study
pSC733	pKNG101-derived allelic exchange plasmid for the generation of chromosomal in-frame Δ*SMDB11_2250* (Δ*paar1*) deletion	This study
pSC1050	pKNG101-derived allelic exchange plasmid for the generation of chromosomal in-frame Δ*SMDB11_1609* (Δ*eagR2*) deletion	This study
pSC706	pKNG101-derived allelic exchange plasmid for the generation of chromosomal in-frame Δ*SMDB11_2263* (Δ*hcp1*) deletion	This study
pSC707	pKNG101-derived allelic exchange plasmid for the generation of chromosomal in-frame Δ*SMDB11_3455* (Δ*hcp2*) deletion	This study
pSC708	pKNG101-derived allelic exchange plasmid for the generation of chromosomal in-frame Δ*SMDB11_3456* (Δ*hcp3*) deletion	This study
pSC737	pKNG101-derived allelic exchange plasmid for the generation of chromosomal in-frame Δ*SMDB11_0927* (Δ*slp*) deletion	This study

pSC739	pKNG101-derived allelic exchange plasmid for the generation of chromosomal in-frame Δ*SMDB11_0927-0929* deletion	This study
pSC727	pKNG101-derived allelic exchange plasmid for the incorporation of a *vgrG1-His* (*SMDB11_2244-His$_6$*) allele at the normal chromosomal location	This study
pSC728	pKNG101-derived allelic exchange plasmid for the incorporation of a *vgrG2-His* (*SMDB11_2276-His$_6$*) allele at the normal chromosomal location	This study
pSC754	pKNG101-derived allelic exchange plasmid for the incorporation of an *ssp4-HA* (*SMDB11_3980-HA*) allele at the normal chromosomal location	This study
pSC671	pKNG101-derived allelic exchange plasmid for the incorporation of a *FLAG-rhs1* (*3xFLAG-SMDB11_2278*) allele at the normal chromosomal location	This study
pSC622	Coding sequence for VgrG1 (SMDB11_2244) in pSUPROM	This study
pSC623	Coding sequence for VgrG2 (SMDB11_2276) in pSUPROM	This study
pSC715	Coding sequence for Hcp1 (SMDB11_2263) in pSUPROM	This study
pSC734	Coding sequence for Paar1 (SMDB11_2250) in pSUPROM	This study
pSC772	Coding sequence for Slp (SMDB11_0927) in pSUPROM	This study
pSC788	Coding sequence for Rhs2 + RhsI2 (SMDB11_1610 + 1611) in pSUPROM	This study
pSC643	Coding sequence for Rhs1 (SMDB11_2278) in pBAD18-Kn	[3]
pSC791	Coding sequence for Rhs1 + RhsI1 (SMDB11_2278 +2278A) in pBAD18-Kn	This study
pSC697	Coding sequence for FLAG-Rhs1 (SMDB11_2278) in pBAD18-Kn	This study
pSC699	Coding sequence for Rhs1^{NT} (SMDB11_2278; amino acids 1-422) in pUT18	This study
pSC700	Coding sequence for Rhs2^{NT} (SMDB11_1610; amino acids 1-363) in pUT18	This study
pSC688	Coding sequence for EagR1 (SMDB11_2277) in pT25	This study
pSC689	Coding sequence for EagR2 (SMDB11_1609) in pT25	This study
pSC048	Coding sequence for TssK (SMDB11_2253) in pUT18	This study
pSC053	Coding sequence for TssK (SMDB11_2253) in pT25	This study
pSC690	Coding sequence for Rhs1$_{NT}$ (SMDB11_2278; amino acids 1-422) in pT25	This study
pSC686	Coding sequence for EagR1 (SMDB11_2277) in pUT18	This study
pSC1056	Coding sequence for EagR2 (SMDB11_1609) in pUT18	This study

References

1. Flyg C, Kenne K, Boman HG (1980) Insect pathogenic properties of *Serratia marcescens*: phage-resistant mutants with a decreased resistance to *Cecropia* immunity and a decreased virulence to Drosophila. J Gen Microbiol 120: 173-181.
2. Murdoch SL, Trunk K, English G, Fritsch MJ, Pourkarimi E, et al. (2011) The opportunistic pathogen *Serratia marcescens* utilizes Type VI secretion to target bacterial competitors. J Bacteriol 193: 6057-6069.
3. Alcoforado Diniz J, Coulthurst SJ (2015) Intraspecies Competition in *Serratia marcescens* Is Mediated by Type VI-Secreted Rhs Effectors and a Conserved Effector-Associated Accessory Protein. J Bacteriol 197: 2350-2360.
4. English G, Trunk K, Rao VA, Srikannathasan V, Hunter WN, et al. (2012) New secreted toxins and immunity proteins encoded within the Type VI secretion system gene cluster of *Serratia marcescens*. Mol Microbiol 86: 921-936.
5. Fritsch MJ, Trunk K, Diniz JA, Guo M, Trost M, et al. (2013) Proteomic identification of novel secreted antibacterial toxins of the *Serratia marcescens* Type VI secretion system. Mol Cell Proteomics 12: 2735-2749.
6. Casadaban MJ, Cohen SN (1979) Lactose genes fused to exogenous promoters in one step using a Mu-lac bacteriophage: in vivo probe for transcriptional control sequences. Proc Natl Acad Sci U S A 76: 4530-4533.
7. Herrero M, de Lorenzo V, Timmis KN (1990) Transposon vectors containing non-antibiotic resistance selection markers for cloning and stable chromosomal insertion of foreign genes in

gram-negative bacteria. J Bacteriol 172: 6557-6567.
8. Grinter NJ (1983) A broad-host-range cloning vector transposable to various replicons. Gene 21: 133-143.
9. Kaniga K, Delor I, Cornelis GR (1991) A wide-host-range suicide vector for improving reverse genetics in gram-negative bacteria: inactivation of the *blaA* gene of *Yersinia enterocolitica*. Gene 109: 137-141.
10. Jack RL, Buchanan G, Dubini A, Hatzixanthis K, Palmer T, et al. (2004) Coordinating assembly and export of complex bacterial proteins. EMBO J 23: 3962-3972.
11. Guzman LM, Belin D, Carson MJ, Beckwith J (1995) Tight regulation, modulation, and high-level expression by vectors containing the arabinose P_{BAD} promoter. J Bacteriol 177: 4121-30.
12. Karimova G, Ullmann A, Ladant D (2001) Protein-protein interaction between *Bacillus stearothermophilus* tyrosyl-tRNA synthetase subdomains revealed by a bacterial two-hybrid system. J Mol Microbiol Biotechnol 3: 73-82.
13. Karimova G, Pidoux J, Ullmann A, Ladant D (1998) A bacterial two-hybrid system based on a reconstituted signal transduction pathway. Proc Natl Acad Sci U S A 95: 5752-5756.
