# Supplementary material for: VgrG and PAAR Proteins Define Distinct Versions of a Functional Type VI Secretion System
Source: PLoS Pathog. 2016 Jun 28;12(6):e1005735. doi: 10.1371/journal.ppat.1005735 (PMC4924876; doi:10.1371/journal.ppat.1005735)
Supplement: S2 Table — (PDF) [file ppat.1005735.s010.pdf]

S2 Table. Oligonucleotide primers for plasmid construction.

Plasmid	Sequence of relevant primers (5'-3')[1]	Description
pSC601	TATATCTAGAATTCTCGCGGCCGATGATG	Forward primer to clone upstream region of SMDB11_2276 in pKNG101 (*Xba*I)
	TATGAAGCTTGTCCAACATGAGAACGTCCTCTG	Reverse primer to clone upstream region of SMDB11_2276 in pKNG101 (*Hind*III)
	TGTAAAGCTTTTCCCGAAATCCTGATGCC	Forward primer to clone downstream region of SMDB11_2276 in pKNG101 (*Hind*III)
	TATAGGGCCCTGGTGTGGATCAGTTCATCATC	Reverse primer to clone downstream region of SMDB11_2276 in pKNG101 (*Apa*I)
pSC602	TATATCTAGAGCCTTTCCCCATTGAGCTG	Forward primer to clone upstream region of SMDB11_2244 in pKNG101 (*Xba*I)
	TATAAAGCTTAAGACGATCCATACTATTCCCCG	Reverse primer to clone upstream region of SMDB11_2244 in pKNG101 (*Hind*III)
	TGTAAAGCTTGCGATCTTCTTGTAAAGGGACTCC	Forward primer to clone downstream region of SMDB11_2244 in pKNG101 (*Hind*III)
	TATAGTCGACATCGCAGCGAGCAATACG	Reverse primer to clone downstream region of SMDB11_2244 in pKNG101 (*Sal*I)
pSC706	TGTGTCTAGATTAACTGCATGCTTGCTTATCACC	Forward primer to clone upstream region of SMDB11_2263 in pKNG101 (*Xba*I)
	TATACTCGAGAATAGCCATAGTTTCTTCACTCTTTATT AAGGG	Reverse primer to clone upstream region of SMDB11_2263 in pKNG101 (*Xho*I)
	TATACTCGAGAACCGCGAAGCTTAATCTTCC	Forward primer to clone downstream region of SMDB11_2263 in pKNG101 (*Xho*I)
	TATAGGGCCCGCGTAGTTTTTGAGGTACTCTTCG	Reverse primer to clone downstream region of SMDB11_2263 in pKNG101 (*Apa*I)
pSC707	TATATCTAGAGCTAAAGCGTTGTTCAACACG	Forward primer to clone upstream region of SMDB11_3455 in pKNG101 (*Xba*I)
	TATAAAGCTTGGCCATTGTCGCCGGATC	Reverse primer to clone upstream region of SMDB11_3455 in pKNG101 (*Hind*III)
	TATAAAGCTTAAAGAGAGCTAAGCGCATTCTGG	Forward primer to clone downstream region of SMDB11_3455 in pKNG101 (*Hind*III)
	TATAGTCGACCACCCGATTATTCGATTCGTG	Reverse primer to clone downstream region of SMDB11_3455 in pKNG101 (*Sal*I)
pSC708	TATATCTAGATTCCGAATAAAAACGACATTACTGTC	Forward primer to clone upstream region of SMDB11_3456 in pKNG101 (*Xba*I)
	TATAAAGCTTTGCCATGATTTTATCACCCG	Reverse primer to clone upstream region of SMDB11_3456 in pKNG101 (*Hind*III)
	TATAAAGCTTAATAAAGAAAGCTAATCACATTACGGT GC	Forward primer to clone downstream region of SMDB11_3456 in pKNG101 (*Hind*III)
	TATAGTCGACTTCAAACTTGAAATCATTAAAGGCATTG	Reverse primer to clone downstream region of SMDB11_3456 in pKNG101 (*Sal*I)
pSC727	TATATCTAGAATCACCGGCAATCAGTCTTTC	Forward primer to clone upstream region of SMDB11_2244 in pKNG101 (*Xba*I)
	TATACTGCAGCAAGAAGATCGCCAGCGC	Reverse primer to clone upstream region of SMDB11_2244 in pKNG101 (*Pst*I)
	TATACTGCAGCATCATCATCATCATCACTAAAGGGACT CCCATGTCGAAG	Forward primer to clone dowstream region of SMDB11_2244, incorporating His_6 tag, in pKNG101 (*Pst*I)
	TATAGTCGACGATCGCAGCGAGCAATACG	Reverse primer to clone downstream region of SMDB11_2244 in pKNG101 (*Sal*I)
pSC728	TATATCTAGAGACTTTATCAACGGCGATCC	Forward primer to clone upstream region of SMDB11_2276 in pKNG101 (*Xba*I)

	TATACTGCAGGGATTTCGGGAACAGCTGATC	Reverse primer to clone upstream region of SMDB11_2276 in pKNG101 (*Pst*I)
	TATACTGCAGCATCATCATCATCATCACTGATGCCGACGCGGCCCTG	Forward primer to clone downstream region of SMDB11_2276, incorporating His_6 tag, in pKNG101 (*Pst*I)
	TATAGGGCCCGCAAAACCGACGATCAGC	Reverse primer to clone downstream region of SMDB11_2244 in pKNG101 (*Apa*I)
pSC733	TATATCTAGACCAGGTCGTCAACCTGTTGC	Forward primer to clone upstream region of SMDB11_2250 in pKNG101 (*Xba*I)
	TGTGACTAGTAAACATAAAAGGCCTCCGCAG	Reverse primer to clone upstream region of SMDB11_2250 in pKNG101 (*Spe*I)
	TATAACTAGTCTGCTTGCGCCCTGATGAC	Forward primer to clone downstream region of SMDB11_2250 in pKNG101 (*Spe*I)
	TATAGGGCCCAGCGATAGGGGTCGTCATCATAG	Reverse primer to clone downstream region of SMDB11_2250 in pKNG101 (*Apa*I)
pSC737	TATAACTAGTTTTCATACGGCGCCTGCC	Forward primer to clone upstream region of SMDB11_0927 in pKNG101 (*Xba*I)
	TATAACTAGTCAGGTGAATTAGTTCGCCTCG	Reverse primer to clone upstream region of SMDB11_0927 in pKNG101 (*Spe*I)
	TATAACTAGTGCCACAATCAAGGGAAGAATATG	Forward primer to clone downstream region of SMDB11_0927 in pKNG101 (*Spe*I)
	TATAGGGCCCATCGCTATCGGCGCCTTTTTC	Reverse primer to clone downstream region of SMDB11_0927 in pKNG101 (*Apa*I)
pSC739	TATAACTAGTTTTCATACGGCGCCTGCC	Forward primer to clone upstream region of SMDB11_0927 in pKNG101 (*Xba*I)
	TATAACTAGTCAGGTGAATTAGTTCGCCTCG	Reverse primer to clone upstream region of SMDB11_0927 in pKNG101 (*Spe*I)
	TATAACTAGTCAGGTGAATTAGTTCGCCTCG	Forward primer to clone downstream region of SMDB11_0929 in pKNG101 (*Spe*I)
	TATAGGGCCCTTCCTGTTCGGCGCCTATGTG	Reverse primer to clone downstream region of SMDB11_0929 in pKNG101 (*Apa*I)
pSC671	TATATCTAGAGGATCTCAACCAGCCTGGC	Forward primer for 1st PCR fragment for overlap PCR to construct 3xFLAG-SMDB11_2278 allele in pKNG101 (*XbaI*)
	CACCGTCATGGTCTTTGTAGTCCATGGCTATTGTTCACTGTGTTCG	Reverse primer for 1st PCR fragment for overlap PCR to construct 3xFLAG-SMDB11_2278 allele in pKNG101
	CGAACACAGTGAACAATAGCCATGGACTACAAAGACCATGACGGTG	Forward primer for 2nd PCR fragment for overlap PCR to construct 3xFLAG-SMDB11_2278 allele in pKNG101
	GCTGCCCAGATCTCAGCCTTGTCATCGTCATCCTTGTAATC	Reverse primer for 2nd PCR fragment for overlap PCR to construct 3xFLAG-SMDB11_2278 allele in pKNG101
	GATTACAAGGATGACGATGACAAGGCTGAGATCTGGGCAGC	Forward primer for 3rd PCR fragment for overlap PCR to construct 3xFLAG-SMDB11_2278 allele in pKNG101
	TATAGGGCCCCGTTGATAAATACTTTACTGGAGC	Reverse primer for 3rd PCR fragment for overlap PCR to construct 3xFLAG-SMDB11_2278 allele in pKNG101 (*ApaI*)
pSC1050	TATATCTAGAGAAGCTGTACTGGTAATGCGC	Forward primer to clone upstream region of SMDB11_1609 in pKNG101 (*XbaI*)
	TATAGTCGACGACATGCGAGGATCCTTTTG	Reverse primer to clone upstream region of SMDB11_1609 in pKNG101 (*SalI*)
	TATAGTCGACGGCGGCCTAACGATGAACTC	Forward primer to clone downstream region of SMDB11_1609 in pKNG101 (*SalI*)

	TATAGGGCCCGATCGGAGACCTTATACAGCCAATC	Reverse primer to clone downstream region of SMDB11_1609 in pKNG101 (*ApaI*)
pSC622	TATAAGATCTATGGATCGTCTTATCATTGCG	Forward primer to clone SMDB11_2244 in pSUPROM (*Bgl*II)
	TATATCTAGATTACAAGAAGATCGCCAGCG	Reverse primer to clone SMDB11_2244 in pSUPROM (*Xba*I)
pSC623	TATATCTAGAATGTTGGACCGCATTATTGC	Forward primer to clone SMDB11_2276 in pSUPROM (*Xba*I)
	TATAGTCGACTCAGGATTTCGGGAACAGC	Reverse primer to clone SMDB11_2276 in pSUPROM (*Sal*I)
pSC715	TATAGGATCCATGGCTATTGATATGTTCCTGAAAGTTG	Forward primer to clone SMDB11_2263 in pSUPROM (*BamHI*)
	TATATCTAGATTAAGCTTCGCGGTTTTCCTTG	Reverse primer to clone SMDB11_2263 in pSUPROM (*XbaI*)
pSC734	TATAGGATCCATGTTTGCCAACAGCCAAATG	Forward primer to clone SMDB11_2250 in pSUPROM (*Bam*HI)
	TATATCTAGATCAGGGCGCAAGCAGCAAC	Reverse primer to clone SMDB11_2250 in pSUPROM (*Xba*I)
pSC772	TATAGGATCCGTGATAATTTTGCCCATACCCTATTTAG	Forward primer to clone SMDB11_0927 in pSUPROM (*Bam*HI)
	TATATCTAGATCATATTCTTCCCTTGATTGTGGC	Reverse primer to clone SMDB11_0927 in pSUPROM (*Xba*I)
pSC697	TATACATATGGACTACAAAGACCATGACGGTG	Forward primer to amplify N-term region of 3xFLAG-SMDB11_2278 from pSC671 to replace the corresponding region in pSC643 (pBAD18-Rhs1) (*Nde*I)
	CCATAATCCCAGAAGCTTTGCG	Reverse primer to amplify N-term region of 3xFLAG-SMDB11_2278 from pSC671 to replace the corresponding region in pSC643 (pBAD18-Rhs1) (*Hin*dIII)
pSC788	TATATCTAGAAAGGACCTTACGATGACCGAAGC	Forward primer to clone SMDB11_1609 and SMDB11_1610 in pSUPROM (*Xba*I)
	TGTGGTCGACTTAAATGGTAACTATCTTATCTTTTGCT ATACCCACAG	Reverse primer to clone SMDB11_1609 and SMDB11_1610 in pSUPROM (*Sal*I)
pSC791	TGTGGAATTCTCTAGAAGAGGATGTAAAATGCAACTA GATACTTATGACGG	Forward primer to clone SMDB11_2278A into pSC643 (*Eco*RI)
	TGTGGAGCTCTTAATAAATTATGTTATTCCTTTTGCAA TATTTTCG	Forward primer to clone SMDB11_2278A into pSC643 (*Sac*I)
pSC699	TATACTGCAGGTAAAGAGGAGGAGCCATGGCTGAGAT CTGG	Forward primer to clone SMDB11_2278 (amino acids 1- 422) in pUT18 (*Pst*I)
	TATAGGATCCGACAGGCGGGAAGGGGTCTG	Reverse primer to clone SMDB11_2278 (amino acids 1-422) in pUT18 (*Bam*HI)
pSC700	TATAGTCGACGTAAAGAGGAGGTACGATGACCGAAGC GG	Forward primer to clone SMDB11_1610 (amino acids 1-363) in pUT18 (*Sal*I)
	TATAGGATCCGAGCTCGGCTGCTCGCC	Reverse primer to clone SMDB11_1610 (amino acids 1- 363) in pUT18 (*Bam*HI)
pSC688	TATAGGATCCAATGGATTACCTTTTCCAGGAAGG	Forward primer to clone SMDB11_2277 in pT25 (*Bam*HI)
	TATAGGTACCCTATTGTTCACTGTGTTCGGTC	Reverse primer to clone SMDB11_2277 in pT25 (*Kpn*I)
pSC689	TATAGGATCCAATGTCCGCTTTATCCCGCTG	Forward primer to clone SMDB11_1609 in pT25 (*Bam*HI)
	TATAGGTACCTTACGCAAAGAGTTCATCGTTAGG	Reverse primer to clone SMDB11_1609 in pT25 (*Kpn*I)
pSC048	TATAGCATGCGTAAAGAGGAGGTGTGATGATGAAAGA TGCTCATAAGG	Forward primer to clone SMDB11_2253 in pUT18 (*Sph*I)

	TATATCTAGAGACCGGTCCGTATGGCTGC	Reverse primer to clone SMDB11_2253 in pUT18 (*Xba*I)
pSC053	TATAGGATCCAATGATGAAAGATGCTCATAAGGTTGTC	Forward primer to clone SMDB11_2253 in pT25 (*Bam*HI)
	TATAGGTACCTTACCGGTCCGTATGGCTG	Reverse primer to clone SMDB11_2253 in pT25 (*Kpn*I)
pSC690	TATAGGATCCAATGGCTGAGATCTGGGCAG	Forward primer to clone SMDB11_2278 (amino acids 1-422) in pT25 (*Bam*HI)
	TATAGGTACCTTACAGGCGGGAAGGGGTC	Reverse primer to clone SMDB11_2278 (amino acids 1-422) in pT25 (*Kpn*I)
pSC686	TATAGCATGCGTAAAGAGGAGGATCCATGGATTACCTTTTCCAGG	Forward primer to clone SMDB_2277 in pUT18 (*Sph*I)
	TATATCTAGAGATTGTTCACTGTGTTCGGTCAAG	Reverse primer to clone SMDB11_2277 in pUT18 (*Xba*I)
pSC1056	TATAGCATGCGTAAAGAGGAGGTCGCATGTCCGCTTTATCC	Forward primer to clone SMDB_1609 in pUT18 (*Sph*I)
	TATATCTAGAGAGGCCGCCCGGTAGCTG	Reverse primer to clone SMDB11_1609 in pUT18 (*Xba*I)
pSC754	Synthetic insert produced by Life Technologies: Comprises 550 bp immediately upstream of the stop codon of SMDB11_3980, followed by an in-frame HA tag (TATCCTTATGATGTTCCTGATTATGCA), the final five codons of SMDB11_3980 (including stop), and then 535 bp downstream of SMDB11_3980.	

[1]Incorporated restriction sites for cloning into the respective vector are underlined.
